# The SMC Loader Scc2 Promotes ncRNA Biogenesis and Translational Fidelity

**DOI:** 10.1371/journal.pgen.1005308

**Published:** 2015-07-15

**Authors:** Musinu Zakari, Rhonda Trimble Ross, Allison Peak, Marco Blanchette, Chris Seidel, Jennifer L. Gerton

**Affiliations:** 1 Stowers Institute for Medical Research, Kansas City, Missouri, United States of America; 2 Universite Pierre et Marie Curie (Paris VI), Paris, France; 3 Department of Biochemistry and Molecular Biology, University of Kansas School of Medicine, Kansas City, Kansas, United States of America; Karolinska Institutet, SWEDEN

## Abstract

The Scc2-Scc4 complex is essential for loading the cohesin complex onto DNA. Cohesin has important roles in chromosome segregation, DSB repair, and chromosome condensation. Here we report that Scc2 is important for gene expression in budding yeast. Scc2 and the transcriptional regulator Paf1 collaborate to promote the production of Box H/ACA snoRNAs which guide pseudouridylation of RNAs including ribosomal RNA. Mutation of *SCC2* was associated with defects in the production of ribosomal RNA, ribosome assembly, and splicing. While the *scc2* mutant does not have a general defect in protein synthesis, it shows increased frameshifting and reduced cap-independent translation. These findings suggest Scc2 normally promotes a gene expression program that supports translational fidelity. We hypothesize that translational dysfunction may contribute to the human disorder Cornelia de Lange syndrome, which is caused by mutations in *NIPBL*, the human ortholog of *SCC2*.

## Author Summary

The structure of chromosomes contributes to the production of RNAs. Chromosome structure is maintained in part by an evolutionarily conserved group of proteins known as structural maintenance of chromosomes proteins. These proteins are loaded onto chromosomes by a second evolutionarily conserved protein complex known as Scc2-Scc4. The Scc2 component is often mutated in a human developmental disorder known as Cornelia de Lange syndrome. We find that Scc2 in budding yeast is important for the production of a group of RNAs known as snoRNAs. These RNAs play a critical role in ribosome production; without them the fidelity of ribosomes suffers. Ribosomes are the molecular machines that translate mRNAs to proteins. Scc2 is also important for the production of the RNA components of ribosomes. Our findings suggest Scc2 normally promotes the production of RNAs that support translational fidelity. We hypothesize that defects in protein synthesis may contribute to Cornelia de Lange syndrome.

## Introduction

Cohesion between sister chromatids generates the force that holds sister chromatids together until the onset of anaphase [1]. Cohesion is generated by cohesin, an evolutionarily conserved multi-subunit protein complex consisting of four core subunits: Smc1, Smc3, the α-kleisin subunit Mcd1/Scc1/Rad21, and the HEAT repeat-containing protein Scc3/SA1 or SA2. The complex forms a ring-like structure that entraps DNA [2, 3]. Smc1 and Smc3 belong to the structural maintenance of chromosome (SMC) ATPase superfamily [1, 4]. This family also includes subunits of the condensin and Smc5/6 complexes. Cohesin and condensin loading are facilitated by Scc2-Scc4 [5–7]. In addition to its role in chromosome segregation, cohesin promotes DNA damage repair [8] and regulates gene expression [9, 10].

It has been proposed that the human ortholog of *SCC2*, *NIPBL*, could regulate gene expression [11]. In budding yeast, Scc2 binds to Pol II transcribed genes encoding ribosomal proteins and small nuclear and nucleolar RNAs (snRNA and snoRNAs), Pol III transcribed genes encoding tRNAs and other noncoding RNAs, as well as pericentric domains [5]. These same regions are bound by condensin [5]. Defects in the association of Scc2 with these highly transcribed regions could potentially affect the expression of these genes. For instance, a recent report suggested Scc2 may help maintain a nucleosome-free region [12], which could potentially promote both SMC complex loading and gene expression. Since Scc2 target genes contribute to translation, a decrease in their expression may negatively affect translation.

Mutations in *SCC2*/*NIPBL* and genes encoding the cohesin subunits result in a developmental syndrome known as Cornelia de Lange syndrome (CdLS). The causative mutations are spread throughout the *NIPBL* gene, most often resulting in a partial loss of function [13]. How mutations in *NIPBL* cause developmental defects remains largely unknown. Examination of cells from affected CdLS probands suggests differential gene expression might cause the developmental defects observed in CdLS patients, rather than chromosome missegregation [14, 15]. Recent work using zebrafish to model CdLS is consistent with the idea that partial loss of function of *nipbl* reduces translation [16], but the molecular mechanisms are unclear.

In this study, we examined how the cohesin loader Scc2 regulates gene expression in budding yeast. We utilized a temperature-sensitive partial loss of function mutation in *SCC2* (*scc2-4*) that has previously been shown to (1) reduce cohesin and condensin association with chromosomes at 37°C [5, 7], (2) delay DSB repair [8], and (3) disrupt nucleolar morphology at 30°C [5]. We found that the mutant protein is expressed at normal levels and displays a normal binding profile at centromeres, but has reduced association with genic regions including ribosomal DNA (rDNA), tDNAs, snoDNAs, and ribosomal protein genes. To examine the biological processes and RNAs regulated by Scc2, we performed RNA sequencing at permissive temperature. Consistent with a previous report [17], we observed differential expression of hundreds of genes. Gene expression signatures suggested both ribosome biogenesis and mitochondrial function would be impacted in the mutant and functional analysis confirms they are both negatively affected. One group of down-regulated genes were the H/ACA snoRNAs, which guide the site-specific pseudouridylaton of rRNAs, tRNAs, mRNAs, and noncoding RNAs [18–20]. The production of H/ACA snoRNAs appears to be facilitated by Scc2-dependent recruitment of the RNA Pol II-associated factor (Paf1) complex. The *scc2-4* mutant showed defective rRNA production and modification with a mild reduction in global protein synthesis. More strikingly, translational fidelity was reduced as shown by decreased internal ribosomal entry site (IRES) usage, increased frameshifting, and decreased resistance to translational inhibitors. Our results in budding yeast strongly suggest that the Scc2 regulated gene expression program promotes translational fidelity.

## Results

### The *scc2-4* mutant protein has reduced binding to genic regions

**Fig 1:**
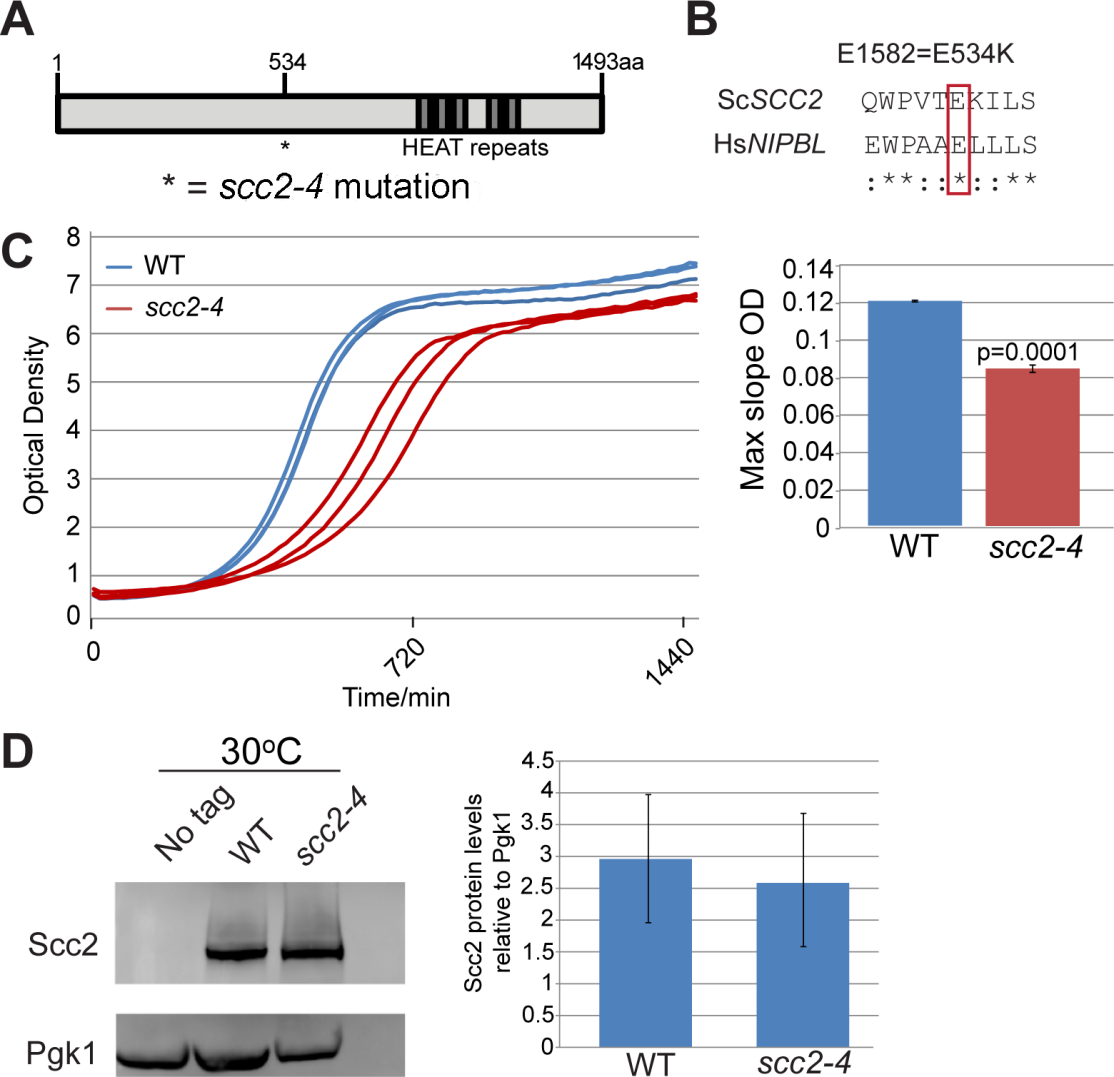
*scc2-4* is a partial loss of function mutation. (A) Scc2 has conserved HEAT repeats in the C terminal domain. (B) The amino acid mutated in *scc2-4* is conserved from yeast to human (E534). (C) A growth curve of the WT and *scc2-4* mutant strains in YPD at 30°C is shown (left). Growth curves were measured using a TECAN machine. The *scc2-4* mutant has a slower maximum growth rate at 30°C compared to the WT, p = 0.0001 (right). (D) The levels of Scc2-Myc were measured by Western blotting (left) and quantified (right, in triplicate with error bars). Pgk1 serves as a loading control. The E534K mutation does not significantly reduce the amount of Scc2 protein at permissive temperature (30°C).

*SCC2* is a large gene encoding a protein with several domains (Fig 1A). Human and budding yeast *NIPBL/SCC2* do not align very well. However, the function of Scc2 in loading SMC complexes is evolutionarily conserved from yeast to humans. Evolutionarily conserved HEAT repeats are located at the C-terminus. By chemical mutagenesis, a temperature sensitive mutant was isolated (E534K), named *scc2-4* [1]. The mutated amino acid is evolutionarily conserved from yeast to human (Fig 1B). The E534K mutation falls in a central region of the protein with unknown function. However, the surrounding amino acids are highly conserved. At 30°C, the *scc2-4* mutant grows more slowly than WT (Fig 1C). Western blot analysis shows that the level of the mutant protein is similar to WT (Fig 1D).

**Fig 2:**
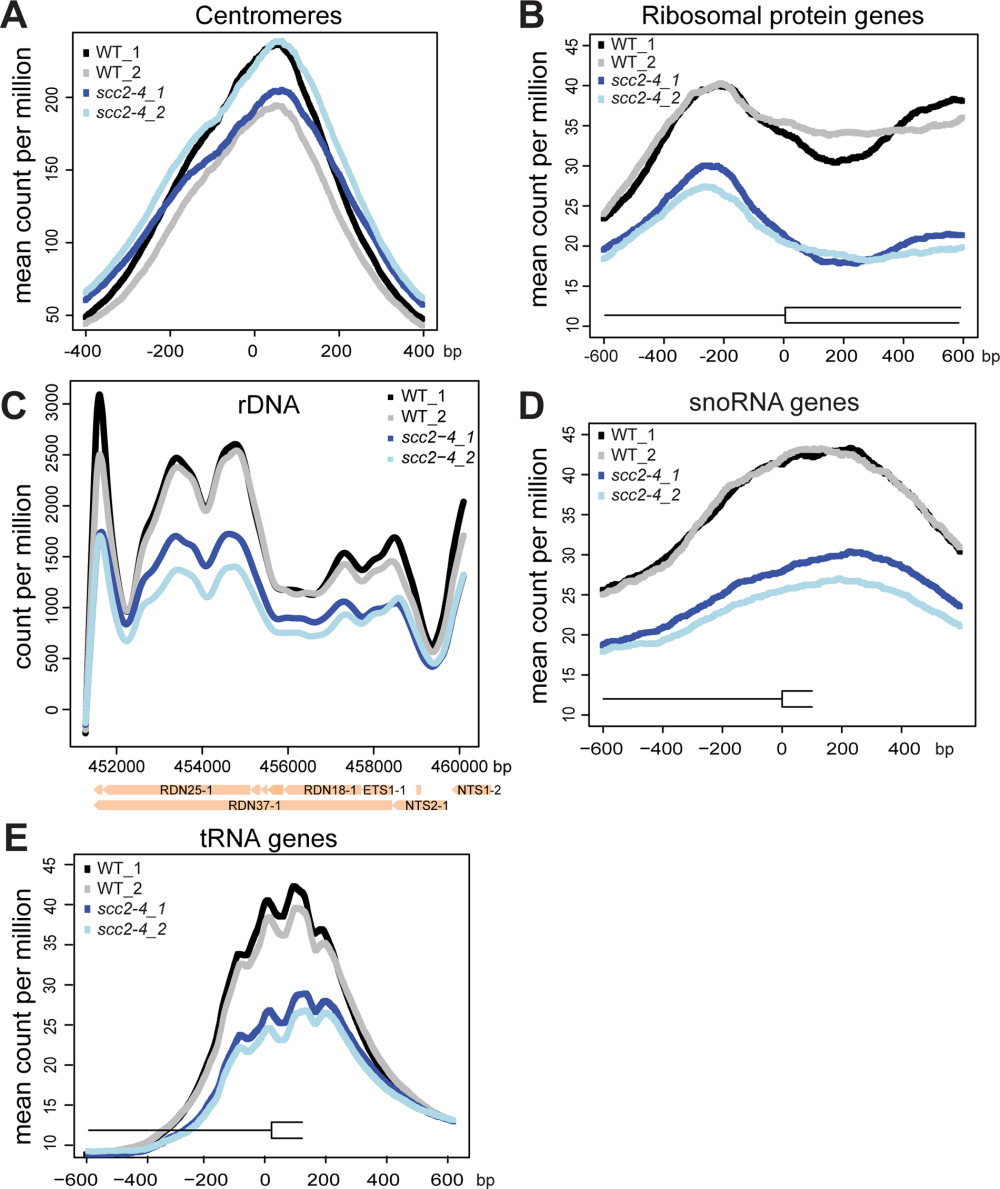
The *scc2-4* mutation compromises the association with genic regions at 30°C. WT and *scc2-4* mutant strains were cultured to mid-log phase (~OD_600_ = 0.5–0.8) in YPD medium. Strains were crosslinked and chromatin extracted for ChIP. Metagene analysis was carried out for Scc2-Myc and Scc2^E534K^-Myc for ChIP seq data. Two biological replicates for each are shown. (A) The *scc2-4* mutation does not affect the association of Scc2 with centromere regions. (B) The mutation reduces the association with ribosomal protein genes (132) (C) rDNA (D) snoDNAs (77) and (E) tDNAs (275).

In order to understand how the mutant Scc2 protein associates with the genome, we performed ChIP-seq. Strains were cultured in YPD+CSM (complete supplement mixture) at 30°C until mid-log phase, fixed in formaldehyde for 2 hrs, and chromatin was harvested for ChIP-seq. The basic pattern of binding for the WT Scc2 protein was similar to previous reports [5, 21]. Enrichment for different regions of the genome was further characterized using metagene plots (Fig 2). On the y-axis is the mean count per million (cpm) and on the x-axis is the position in base pairs. No apparent difference in centromere enrichment was observed for Scc2^E534K^ when compared to the WT (Fig 2A). In contrast, we observed reduced enrichment for rDNA, snoDNAs (Box H/ACA and Box C/D), tDNAs, and the small and large ribosomal protein genes in the Scc2^E534K^ ChIP (Fig 2). Thus, the mutation appears to compromise genic association without compromising centromere association.

### Hundreds of genes are differentially expressed in the *scc2-4* mutant

**Fig 3:**
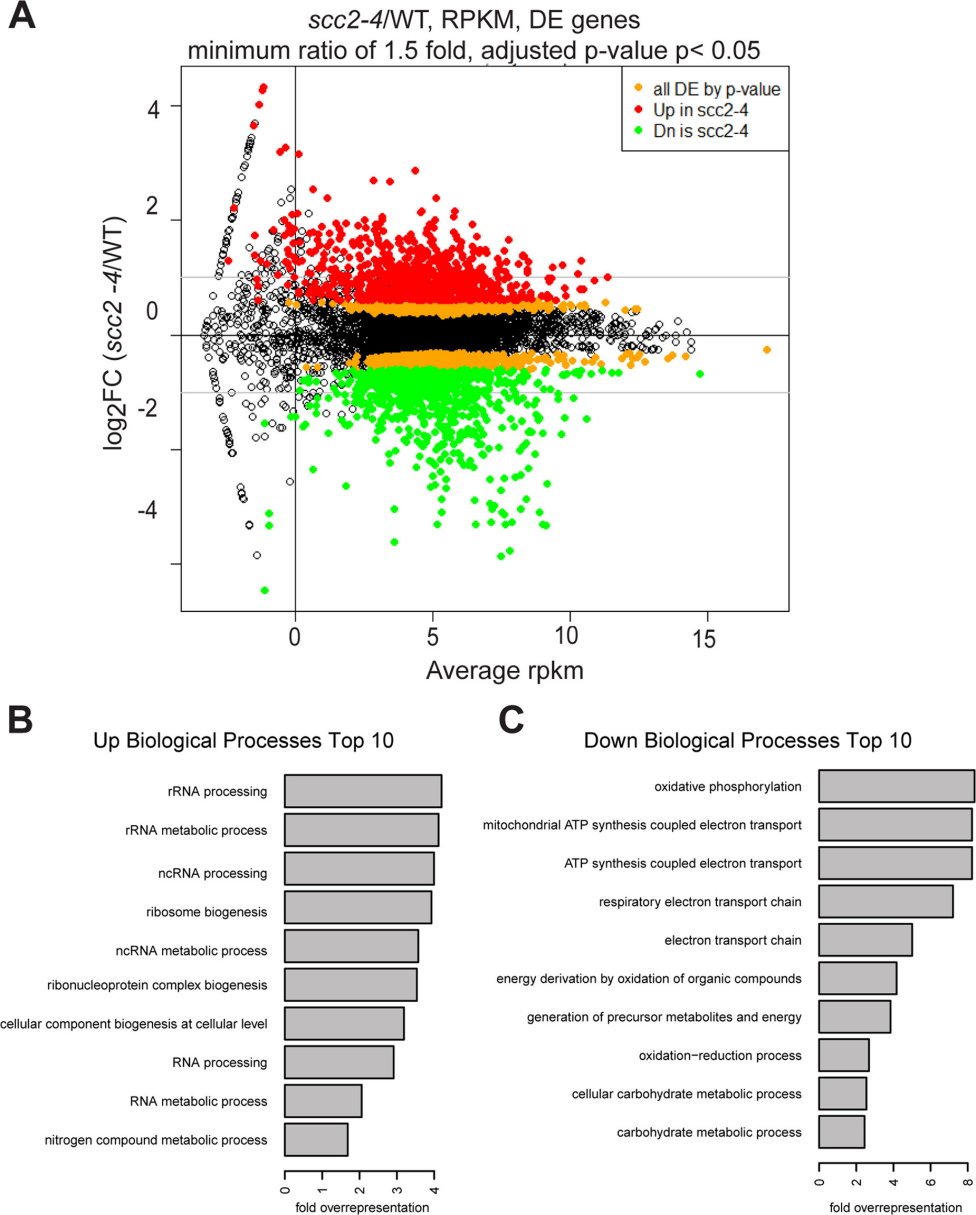
Hundreds of genes are differentially expressed in the *scc2-4* mutant compared to WT at 30°C in YPD. (A) Gene expression values (mean of triplicate samples) are shown in MA plot. Differentially expressed genes (adjusted p-value <0.05) were colored in orange, and then colored red or green after a minimum fold change cut-off of 1.5 was applied. There are 2644 genes differentially expressed genes in the *scc2-4* mutant, with 1285 up-regulated and 1359 down-regulated. Applying a fold-change cutoff of 1.5 (corresponding to an absolute log_2_ value of 0.6) returns a more reasonable number of genes for GO analysis and general comparison. (B) GO term analysis for the up-regulated genes shows enrichment for genes important for RNA processing/metabolism and ribosome biogenesis. (C) GO term analysis for down-regulated genes shows enrichment for genes important for biological processes such as oxidative phosphorylation, electron transport chain, and carbohydrate metabolic processes.

To investigate the biological processes regulated by Scc2, RNA sequencing was performed for WT and the *scc2-4* mutant. Each strain was grown in triplicate, at 30°C in YPD+CSM to mid- log phase. The distribution of gene expression can be viewed in the MA plot, defined as a plot of log-intensity ratios (M-values) versus log-intensity averages (A-values) (Fig 3A). The log_2_ ratio of *scc2-4*/WT is shown on the y-axis and the geometric mean of the reads per kilobase of transcript per million reads mapped (RPKM) values on the x-axis. Using an adjusted *p-value* of 0.05 as criteria, there are 2644 differentially expressed genes, with 1285 up-regulated and 1359 down-regulated in *scc2-4* mutant. Further applying a fold change threshold of 1.5 (corresponding to an absolute log_2_ value of 0.6) returns 823 up-regulated and 760 down-regulated genes for GO analyses and general comparisons. This is many more genes than are bound by Scc2, suggesting that many of the changes in gene expression are due to indirect effects. GO term analyses for up-regulated genes shows enrichment for genes involved in ribosome biogenesis and rRNA processing (Fig 3B and S1A and S1B Fig). The upregulation of ribosomal protein genes (RPs) and the processome in general suggests these mRNAs are not rate limiting in the *scc2-4* mutant for ribosome biogenesis, although ribosome assembly does appear to be affected (see below). This upregulation is in direct contrast to our previous analysis of gene expression in the cohesin acetyltransferase mutant (*eco1-W216G*), which shows downregulation of these gene groups [22]. GO term analysis for the down-regulated genes shows enrichment for genes required for oxidative phosphorylation (Fig 3C and S1C and S1D Fig).

We further explored specific aspects of the gene expression profile in the *scc2-4* mutant. The downregulation of genes involved in oxidative phosphorylation suggested the mutant might be hypersensitive to a drug that inhibits mitochondrial function, such as chloramphenicol which blocks protein synthesis by the mitochondrial ribosome. Consistent with their respective gene expression profiles, the *scc2-4* mutant, but not the *eco1-W216G* mutant, showed very poor growth on plates with a sublethal dose of chloramphenicol (S2 Fig).

Another group recently published the gene expression profile of the *scc2-4* mutant [17]. However, these experiments were conducted at non-permissive temperature whereas our experiments were all conducted under permissive conditions. The two data sets have some differences, as expected, but the gene expression profile from Lindgren et al suggests mitochondrial function and translation would be negatively impacted in the *scc2-4* mutant, although this was not tested. However, they did show that cohesin binding locations were not significantly affected in the mutant background, helping to rule out this effect as an explanation for the differential gene expression.

Scc2 binds to genes that encode RNA components of ribonucleoprotein complexes such as *NME1*, a component of RNAse MRP that modifies ribosomal RNA, *SCR1*, part of the signal recognition complex, and *SNR6*, the U6 component of the spliceosome. The mutant protein binds less well to these regions, correlating with significantly lower expression in the mutant (p<0.05). Given the reduction in U6, we decided to analyze splicing in the mutant compared to WT. By measuring splicing efficiency (computing the mean nucleotide coverage over every spliced junction divided by the mean nucleotide coverage for reads falling within the exonic splicing unit), we were able to detect a modest but reproducible defect in splicing (S3 Fig). As a control we used the *eco1-W216G* mutant, where we did not find a splicing defect (S3 Fig).

Analysis of the correlation between genome-wide enrichment of Scc2-bound sequences and gene expression revealed contrasting findings. While loss of binding of Scc2 correlated with lower gene expression at certain regions of the genome (e.g. Box H/ACA, some Box C/D snoDNAs), there was increased expression at RP genes, and no correlation at other regions. Overall the gene expression pattern is likely the product of a combination of direct effects from changes in Scc2 binding and indirect effects. It is also possible that Scc2 may play different roles in gene expression at different groups of genes.

### The *scc2-4* mutant has defects in rRNA production and ribosome distribution

**Fig 4:**
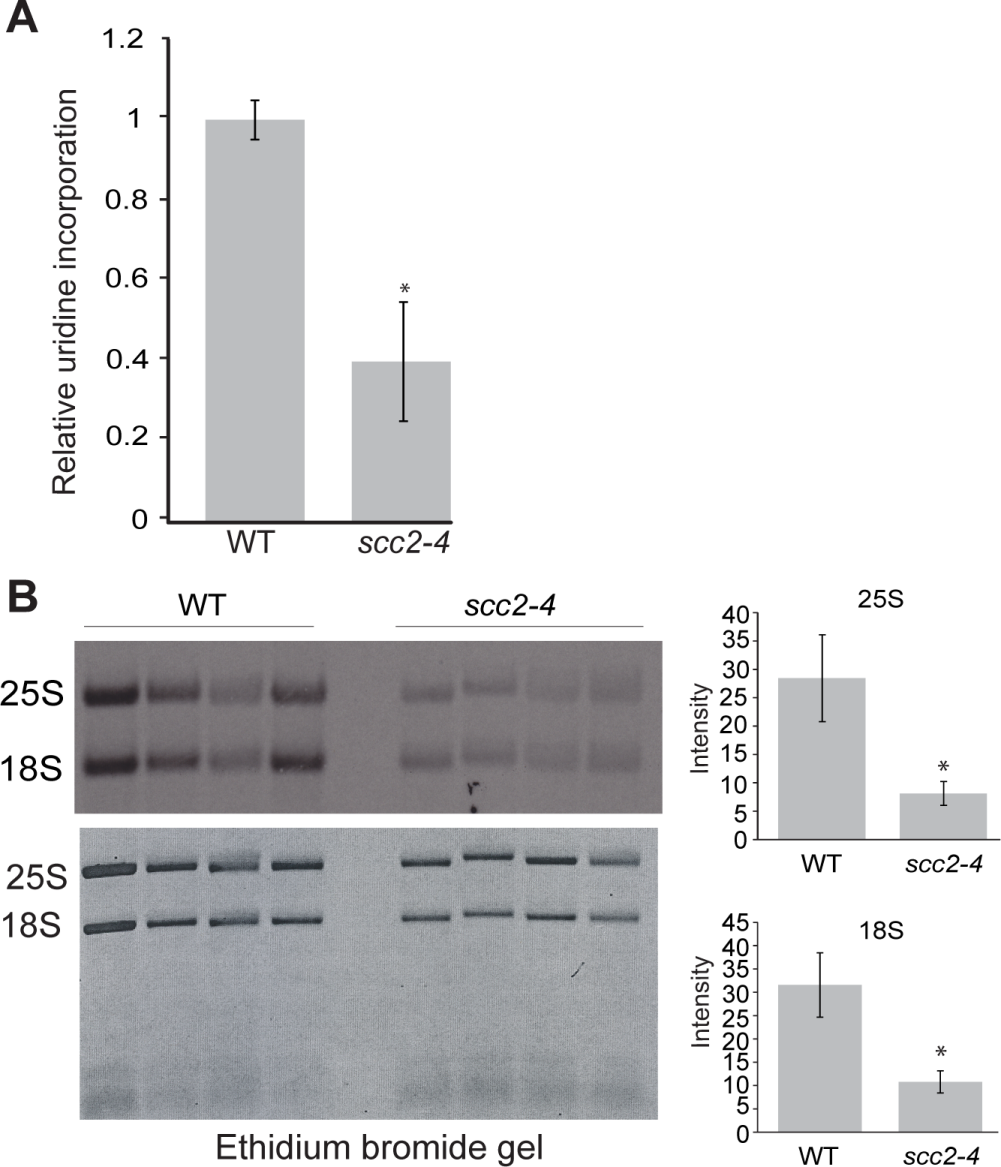
Ribosomal RNA production is compromised in the *scc2-4* mutant. (A) The RNA synthesis rate was examined using ^3^H-uridine incorporation in WT and *scc2-4* mutant strains. Strains were grown in triplicate in SD-ura medium with minimal uracil to an approximate OD_600_ of 0.3. ^3^H-uridine was added for 5 minutes and incorporation was quantified, averaged, and expressed relative to WT. Standard error bars are indicated for n = 4. The difference between WT and mutant was significant at p<0.0001. (B). WT and *scc2-4* mutant strains were grown in SD-met at 30°C into mid-log phase. Equal numbers of cells were pulse-labeled with ^3^H-methylmethionine for 5 min, followed by RNA extraction and electrophoresis of the RNA on a formaldehyde gel. The gel was photographed following ethidium bromide staining (bottom). RNA was transferred to a membrane and detected by autoradiography (top). 25S and 18S rRNA were cut from the membrane, quantified with a scintillation counter, and the average is expressed relative to WT (p<0.05). p-values were calculated using a student two-tailed t-test. Standard error is indicated for n = 4.

The reduced enrichment for the ribosomal DNA repeats in the *scc2-4* mutant (Fig 2C), combined with our previous work demonstrating that cohesion is required to form a nucleolus [23] and produce normal levels of ribosomal RNA [22], drove us to examine rRNA production and nucleolar morphology in the *scc2-4* mutant. rRNA synthesis was measured by pulse-labeling cells for 5 min with ^3^H-uridine. This approach is a reliable method for estimating RNA Pol I activity since most RNA synthesis during exponential growth is Pol I derived. Incorporation of uridine into total RNA was reduced by approximately 3-fold in the *scc2-4* mutant (Fig 4A), consistent with the observed decrease in growth rate in the *scc2-4* mutant. rRNA production was further investigated by pulse-labeling cells with ^3^H-methylmethionine for 5 min, chased with excess cold methionine for 5 min and extracting RNA. Incorporated ^3^H was quantified in 25S and 18 rRNAs (Fig 4B). rRNA is methylated cotranscriptionally in yeast, making this approach a reliable method for quantifying the production of the methylated forms of the 25S and 18S rRNAs [24]. By this method, we also observed a 3.6-fold reduction in the production of 25S rRNA (Fig 4B, right) and a 4-fold reduction in 18S rRNA production (Fig 4B, bottom right). We further examined the processing of the initial rRNA transcript into the 25S and 18S forms over time. We found no defect in the processing rate for 25S and 18S rRNAs in *scc2-4* mutant (S4 Fig). In summary, the *scc2-4* mutant has reduced rRNA production.

Nucleolar morphology has been reported to be aberrant in the *scc2-4* mutant based on visualization of the nucleolar protein Net1 fused to GFP [5]. We extended this observation using electron microscopy (S5 Fig). The dense staining nucleolar material did not adopt the normal crescent shaped structure in the mutant, consistent with the idea that reduced binding of Scc2 and reduced loading cohesin and/or condensin at the rDNA could result in a failure to gather the rDNA repeats into a normal nucleolar structure.

**Fig 5:**
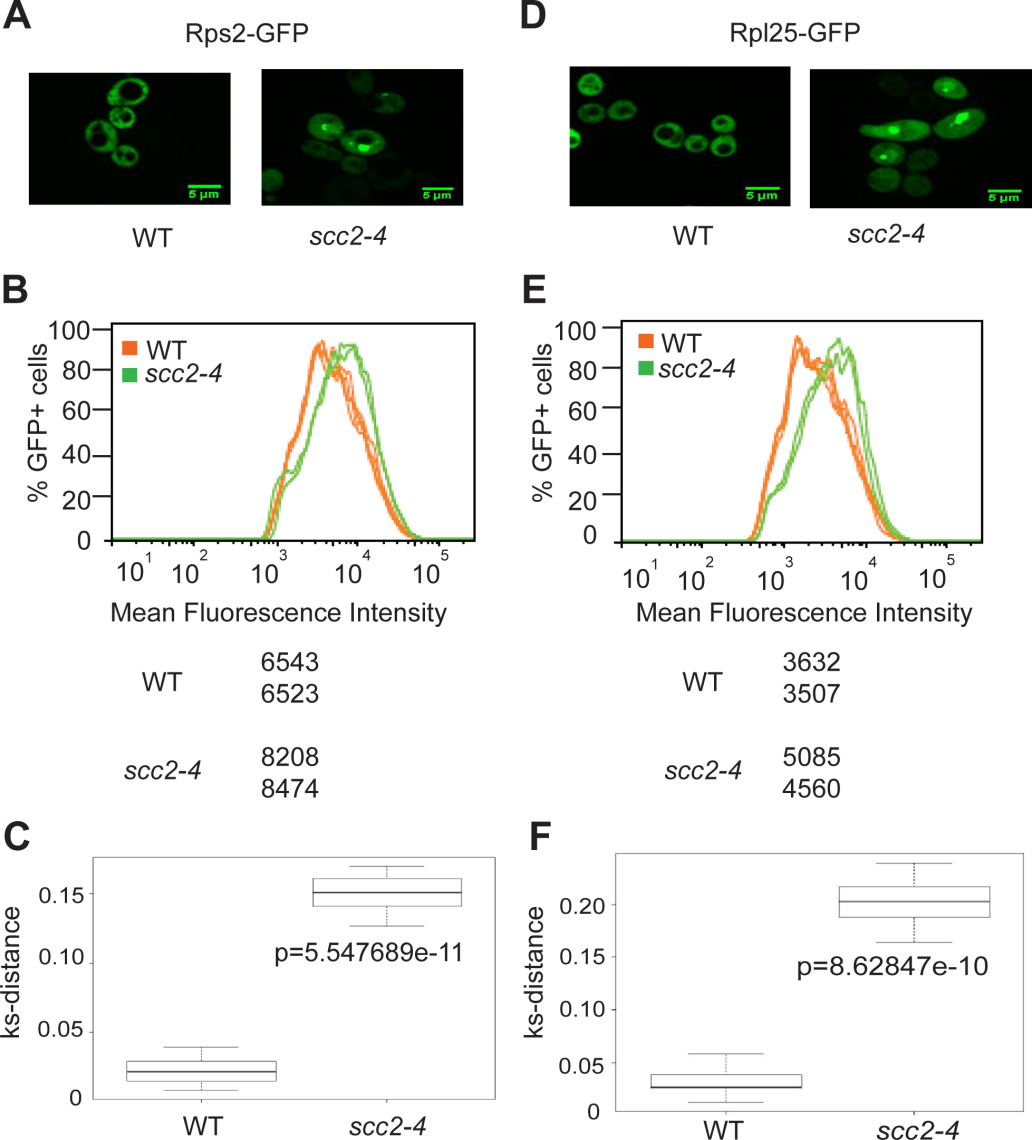
Ribosome protein distribution is compromised in the *scc2-4* mutant. Protein components of the small (Rps2) and large (Rpl25) ribosomal subunits were tagged with GFP and imaged. Representative images are shown for WT and mutant strains (A and D). Using flow cytometry, the peak GFP intensity was quantified for independent biological replicates (B and E). At least 10000 cells were examined per replicate. A KS-distance was calculated from cumulative distribution frequency curves of the fluorescence which allows us to determine statistical significance (C and F) (p<0.0001).

Ribosomes are partially assembled in the nucleolus and transported into the nucleus and then cytoplasm to be fully assembled and engaged in translation. Since mutation in *SCC2* results in differential expression of messages involved in ribosome biogenesis, including ribosomal protein genes and genes involved in processing and assembling ribosomes, we further investigated ribosome production in the *scc2-4* mutant. GFP tagged protein components of the large (Rpl25) and small (Rps2) ribosomal subunits were expressed in the WT and *scc2-4* mutant strains and examined by microscopy. Rather than the normal even distribution in the cytoplasm, we observed accumulation of ribosomal proteins in the *scc2-4* mutant in the nucleus/nucleolus that suggested that ribosome assembly/export was affected (Fig 5A and 5D). Using flow cytometry, we found higher mean fluorescence for both 40S and 60S reporters in the *scc2-4* mutant (Fig 5B and 5E). We further analyzed the data by generating a cumulative distribution function for each sample, calculating the distance between samples, and testing for statistical significance using the Kolmogorov—Smirnov (KS) test, see Materials and Method and [22] (Fig 5D and 5F). The KS test was statistically significant for both the 40S and 60S subunit reporters in the *scc2-4* mutant compared to WT. This analysis suggests ribosome assembly may be deficient in the mutant. The defects in nucleolar morphology, rRNA production, and ribosome distribution in the *scc2-4* mutant are quite similar to those reported for the cohesin acetyltransferase mutant, suggesting cohesion is important for these aspects of nucleolar structure and function.

### Downregulation of H/ACA snoRNAs inhibits site-specific pseudouridylation in the *scc2-4* mutant

**Fig 6:**
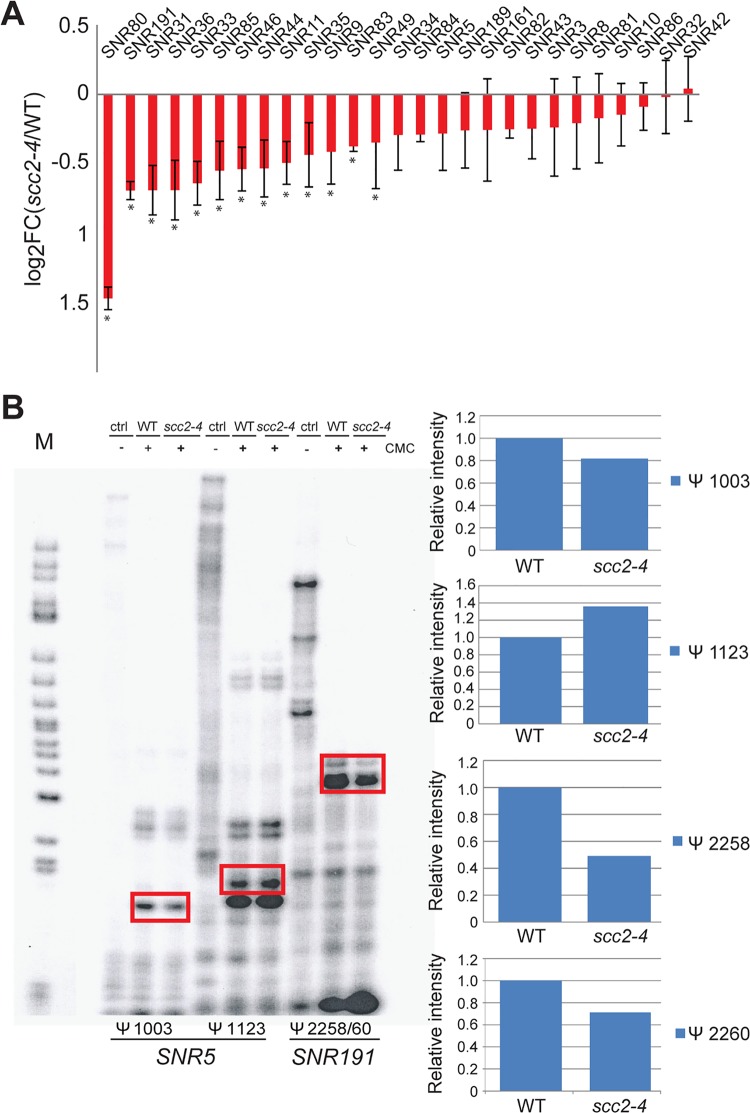
Pseudouridylation of rRNA is reduced in the *scc2-4* mutant. (A) The Box H/ACA snoRNAs that guide sequence-specific pseudouridylation are down-regulated in the *scc2-4* mutant. The error bars represent the standard deviation from triplicate samples and the asterisks indicate statistical significance at an adj p<0.05. (B) Reverse transcription with primers corresponding to residues Ψ 1003 and Ψ1123 for *SNR5* and residues Ψ2258 and Ψ2260 for *SNR191* was performed. Samples were treated with or without CMC, exposed to pH 10.4 for 4 hrs and reverse transcribed. The rectangles indicate the bands quantified to the right. Pseudouridylation assays were performed at least two times; a representative experiment is shown.

One notable group of Scc2 bound genes that was down-regulated in the *scc2-4* mutant was the box H/ACA snoRNA genes (Fig 6A). Some box C/D RNAs were also downregulated, but not all. Box H/ACA snoRNA genes stand alone whereas box C/D snoRNA genes lie in the introns of ribosomal protein genes which may make them subject to the regulation of their host genes. The differential expression of snoRNAs was not observed in the *eco1-W216G* mutant [22]. These small nucleolar rRNAs guide site-specific modification of rRNAs and other RNAs. Box C/D RNAs guide the methylation of RNAs in the context of a ribonucleoprotein particle. The box H/ACA snoRNAs are also part of ribonucleoprotein particles (snoRNPs) along with the essential proteins (Nhp2, Cbf5, Nop10 and Gar1) that together catalyze site-specific pseudouridylation of RNAs. Pseudouridylation is important for RNA stability and interactions with other RNAs and proteins [25]. Mutation of the *CBF5* component of this snoRNP leads to reduced pseudouridylation of rRNA and reduced translational fidelity [26, 27]. These defects are thought to contribute to cases of dyskeratosis congenita caused by mutations in *DKC1*, the human ortholog of *CBF5*.

Downregulation of box H/ACA snoRNAs could reduce pseudouridylation of RNAs. To test this idea, rRNA was isolated from WT and the *scc2-4* mutant and the distribution of pseudouridines was examined by the CMC-primer extension method. Mapping of pseudo-sites on rRNA revealed downregulation of pseudouridylation at positions Ψ1003 (guided by *SNR5*) and Ψ2258/60 (guided by *SNR191*) (Fig 6B). Reduced pseudouridylation may derive from the reduced expression of Box H/ACA snoRNAs observed in the *scc2-4* mutant.

### Paf1 and Scc2 show co-dependent recruitment at snoDNAs

**Fig 7:**
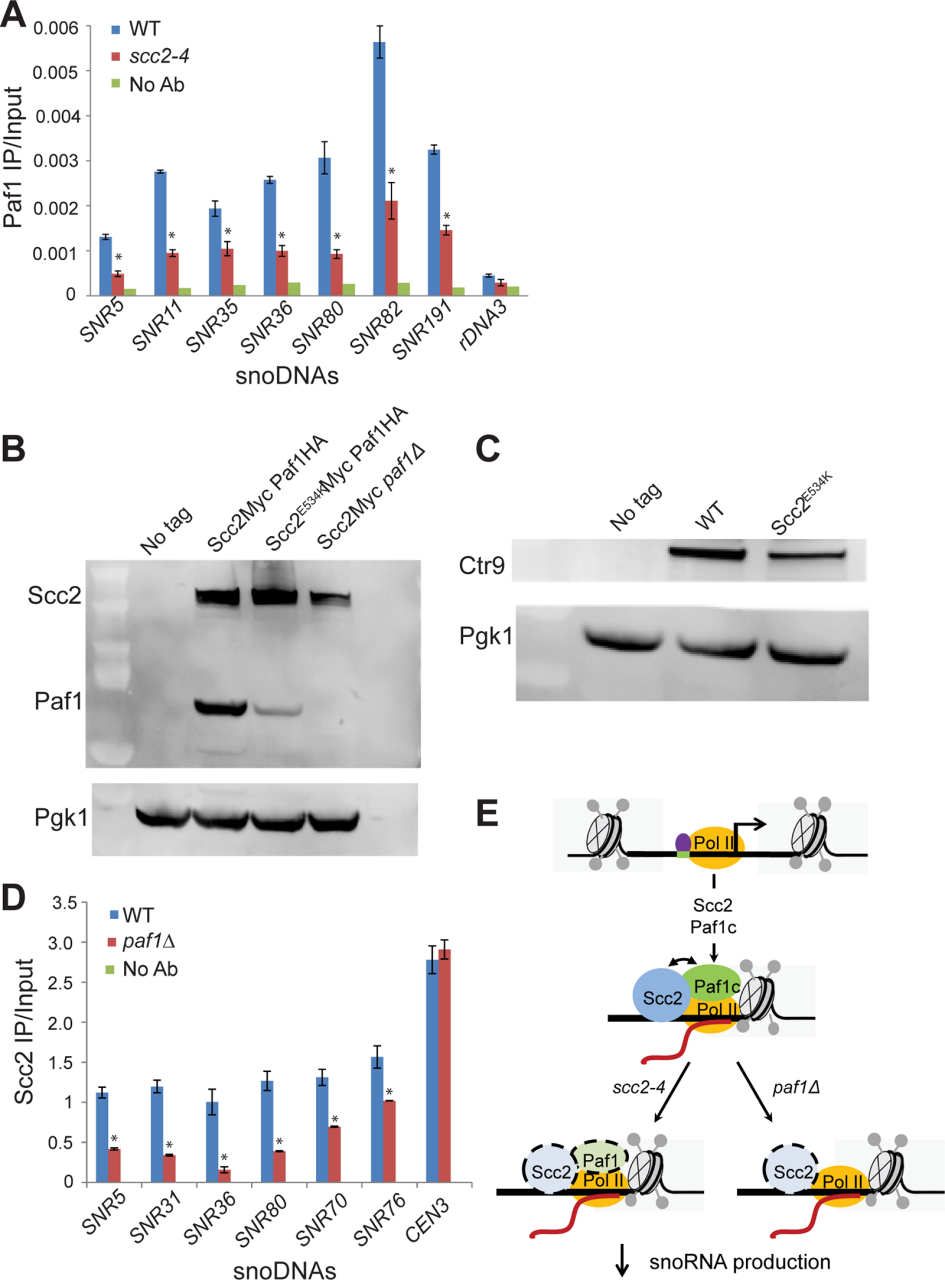
Scc2 and Paf1 recruitment at snoDNAs is co-dependent. (A) WT, *scc2-4*, and *paf1Δ* mutant strains were cultured in YPD medium to mid-log phase (~ OD_600_ = 0.5–0.8). Strains were crosslinked and chromatin extracted for ChIP. ChIP/qPCR analysis was carried out for Scc2-Myc and Scc2^E534K^-Myc and Scc2-Myc *paf1Δ*. ChIP performed without the addition of primary antibody served as a negative control. ChIP experiments were performed at least three times. p-values were calculated using a student t-test. Standard error bars are indicated for n = 3. Values different from the WT are indicated by an asterisk (p<0.05). qPCR analysis shows reduced enrichment of indicated snoDNAs in mutant relative to WT in Paf1 ChIP (α-HA antibody, 12CA5, Roche). (B) Western blot analysis shows that the protein level of Paf1 is reduced in the *scc2-4* mutant. Scc2 levels also appear reduced in the *paf1Δ* strain. Pgk1 served as the loading control. (C) Western blot analysis shows that the protein level of Ctr9 is reduced in the *scc2-4* mutant. (D) qPCR analysis shows reduced enrichment of snoDNAs in the Scc2-Myc ChIP in the *paf1Δ* mutant strain relative to WT (α-Myc antibody, 9B11, Cell signaling). (E) A cartoon model for the co-recruitment of Scc2 and Paf1 at snoDNAs is shown. The Tbf1 transcription factor is shown in purple, RNA Pol II in yellow, and Scc2 and Paf1c in blue and green, respectively.

snoRNAs are transcribed by RNA polymerase II. The Tbf1 transcription factor has been identified at snoRNA genes [28]. We examined whether the enrichment for snoRNA genes was compromised in ChIPs performed for either of these two baits in the *scc2-4* mutant. The qPCR signal for snoRNA genes for both Tbf1 and RNA Pol II (all forms) is similar in WT and *scc2-4* mutant samples (S6 Fig), suggesting defective localization of these proteins cannot account for the lower levels of snoRNAs observed in the mutant. The Paf1 complex, which is comprised of Paf1, Ctr9, Leo1, Rtf1, and Cdc73, is important for transcription elongation. [29–31]. The Paf1 complex has been shown to contribute to the production of snoRNAs [29, 32]. To assess whether Paf1 recruitment was affected, we tagged Paf1 at its endogenous locus and examined the enrichment for snoRNA genes by ChIP. In contrast to RNA Pol II and Tbf1, enrichment for snoDNAs (Box C/D and H/ACA snoRNAs) in the Paf1 ChIP was significantly reduced in the *scc2-4* mutant (Fig 7A). In addition to the snoDNAs, enrichment for rDNA and RP genes was also reduced in Paf1 ChIP in the *scc2-4* mutant (S7 Fig). Given the global decrease in Paf1 recruitment, we next asked about mRNA and protein levels of Paf1 in the *scc2-4* mutant. While mRNA levels for the subunits of the Paf1 complex were not significantly affected in our RNA seq data, we observed less Paf1 protein in the *scc2-4* mutant by Western blot (Fig 7B). The protein level of Ctr9, another member of the Paf1 complex was also reduced (Fig 7C), implying that the *scc2-4* mutation compromises the steady state level of Paf1 complex subunits. Interestingly, deletion of *PAF1* caused a reduction in protein levels of Scc2 (Fig 7B). Therefore, the simplest explanation for the decreases in ChIP/qPCR signal is that Paf1 and Scc2 depend on each other for stable expression.

The Paf1 complex has previously been shown to promote transcription elongation by RNA Pol I [24]. We explored whether Scc2 recruitment to snoDNAs, rDNA, and RP genes was affected in the absence of Paf1. Deletion of *PAF1* resulted in reduced enrichment for snoDNAs in Scc2 ChIP (Fig 7D), implying that Scc2 and Paf1 are dependent on each other for full recruitment at these genes. While enrichment for the rDNA and RP genes was compromised in the Paf1 ChIP in the *scc2-4* mutant (S7A and S7B Fig), deletion of *PAF1* did not affect enrichment for these loci in the Scc2 ChIP (S7C and S7D Fig), suggesting that the low recruitment of Scc2^E534K^ cannot be attributed to less Paf1. In addition, *PAF1* deletion did not compromise Scc2 binding to other Paf1 target genes, implying that the requirement of Paf1 for Scc2 recruitment was unique to snoDNAs. These results suggest that Paf1 and Scc2 cooperate uniquely in their co-recruitment to snoDNAs (Fig 7E).

### The *scc2-4* mutant has poor translational fidelity

Since mutation of *SCC2* affects rRNA biogenesis, we examined actively translating ribosomes by polysome analysis. Polysome analysis of WT and *scc2-4* mutant strains showed nearly identical profiles (S8A Fig). To more carefully investigate the effects of the *scc2-4* mutation on global protein synthesis, ^35^S-incorporation was examined. A mild reduction in protein synthesis in the *scc2-4* mutant was observed (S8B Fig). Consistently, the mutant failed to grow on plates with a sublethal concentration of cycloheximide, an inhibitor of protein translation (S8C Fig).

**Fig 8:**
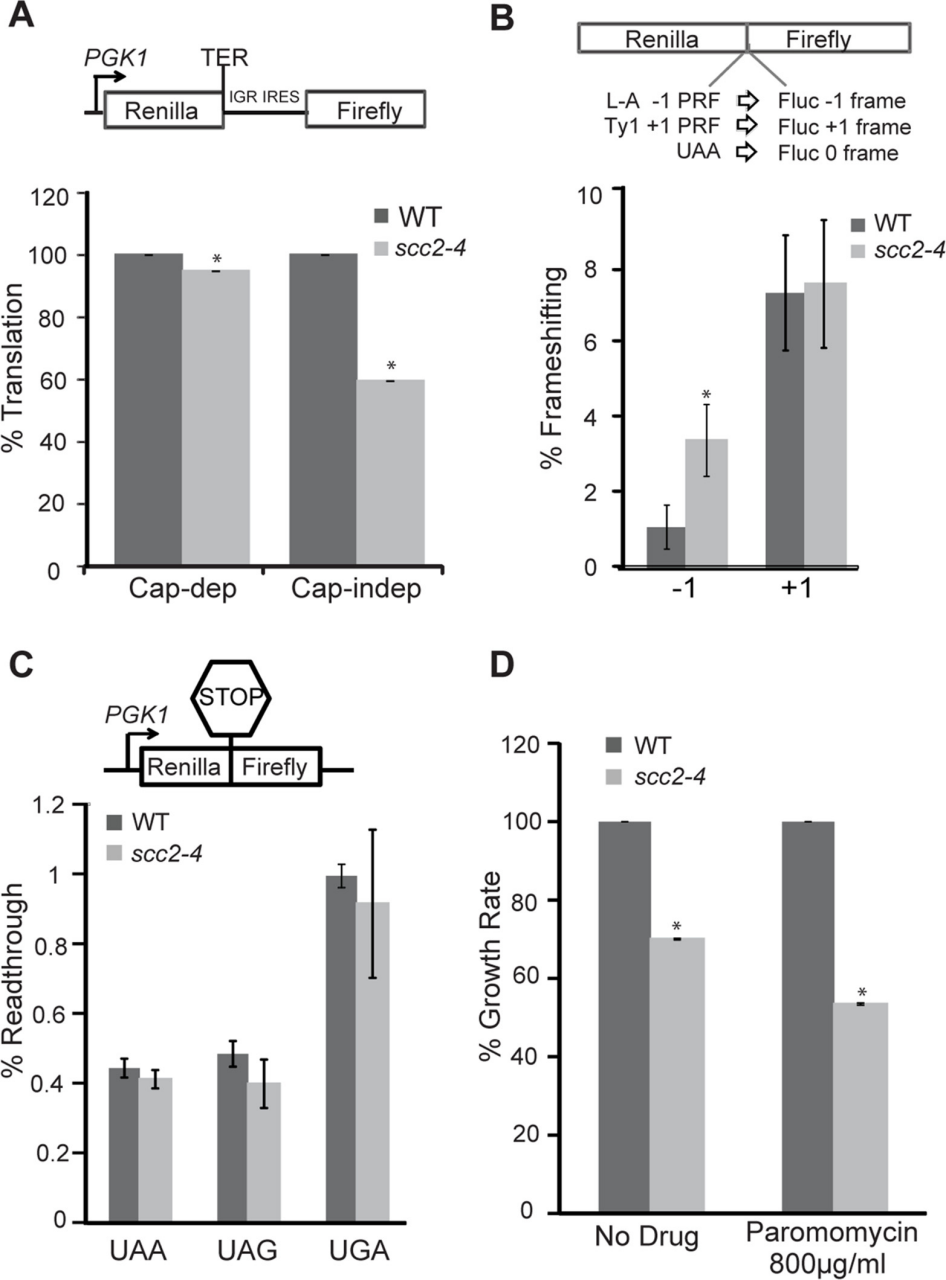
Translational fidelity is reduced in the *scc2-4* mutant. Dual luciferase reporters were used to measure translation. (A, top) Schematic diagram of CrPV IGR IRES containing reporter. Renilla luciferase is translated by a cap-dependent mechanism, and firefly luciferase synthesis requires cap-independent initiation mediated by the IRES. While cap-dependent translation is mildly impaired (p = 0.01), IRES-dependent translation is more strongly inhibited in the *scc2-4* mutant (p = 0.001). (B, top) A dual luciferase reporter was used to monitor -1 frameshifting mediated by sequence derived from the yeast L-A virus, and +1 frameshifting promoted by the yeast Ty1 sequence. In-frame renilla luciferase translation serves as a normalization control, and efficiencies were determined as previously described (Landry et al., 2009) The mutant shows an increase in -1 frameshifting. (C). Readthrough efficiency for three different stop codons (UAA, UAG, UGA) was measured for the WT and *scc2-4* strains. The translation of renilla luciferase serves as a normalization control. (D) The maximal growth rates of the WT and *scc2-4* mutant strains were compared, with the WT growth rate set to 100%. Strains were grown in YPD or YPD with paromomycin. p-values were calculated using student two-tailed t-test (asterisk indicates p<0.05). Standard error is indicated for at least three independent measurements.

We hypothesized that a downregulation of box H/ACA snoRNAs, as observed in the *scc2-4* mutant, might phenocopy a *CBF5* mutant. Mutations in *CBF5* show limited effects on translational efficiency, but significant effects on translational fidelity [26]. We decided to examine translational fidelity in the *scc2-4* mutant. We first tested the ability of the mutant to translate IRES-dependent mRNAs. IRES elements are used by both viral and endogenous genes for cap-independent translation initiation [27]. The Cricket Paralysis virus (CrPV) IRES has been shown to be active in both yeast and mammalian cells [26, 33] and able to initiate translation by directly recruiting ribosomes to the initiation sites without initiation factors. Defects in ribosome biogenesis can impair cap-independent translation initiation directed by CrPV IGR IRES elements [26, 33] and in cellular RNAs that utilize the IRES mechanism [27]. We monitored translation initiation from the CrPV IGR IRES elements using a dual luciferase reporter which is able to measure translation in vivo in yeast cells (Fig 8). While cap-dependent translation was very weakly reduced in the *scc2-4* mutant, consistent with the results of ^35^S-methionine incorporation (S8B Fig); IRES usage was reduced by more than 40% in the *scc2-4* mutant when compared to WT (Fig 8A), implying that Scc2 promotes cap-independent translation.

Faithfully maintaining the translational reading frame is one of the important functions of ribosomes. Viral mRNA signals that can induce ribosomes to shift frame by one base in either -1 or +1 direction have been tremendously useful for examining translational fidelity [26, 34]. We examined frameshifting in the *scc2-4* mutant. Dual luciferase reporters able to detect -1 frameshifting (L-A) or +1 frameshifting (Ty1) were employed. The *scc2-4* mutant had an approximately 3-fold increase in L-A mediated -1 frameshifting compared to WT (3.4% to 1.1%; p = 0.006) (Fig 8B). However, no change in +1 frameshifting was observed. We further examined the efficiency of stop codon recognition using dual luciferase reporters (Fig 8C) in the *scc2-4* mutant. The *scc2-4* mutant was able to recognize stop codons as efficiently as WT.

Since *paf1Δ* mutants also have defects in snoRNA expression, we examined whether a *paf1Δ* mutant had defects in translational fidelity. Translation initiation was examined by transforming the CrPV IGR IRES dual luciferase construct in a *paf1Δ* mutant. Similar to the *scc2-4* mutant, use of the IRES sequence to initiate translation was severely compromised in the *paf1Δ* mutant (S9A Fig). We also observed a significant increase in -1 frameshifting in the *paf1Δ* mutant when compared to the WT strain (S9B Fig). However, +1 frameshifting was unaffected. Overall the *paf1Δ* and *scc2-4* mutants show deficits in translational fidelity similar to those caused by mutation of *CBF5* [26].

Changes in ribosome-tRNA interactions can affect translational fidelity and therefore influence the sensitivity of yeast to translational inhibitors. Translational inhibitors that specifically bind to ribosomes serve as useful tools for examining changes in ribosome function. Anisomycin, which inhibits -1 frameshifting by interfering with binding of aa-tRNA to the A-site, was used to probe for defects in this region of the ribosome [35]. Similarly, paromomycin, which promotes translational misreading, and increases programmed -1 ribosomal frameshifting in a *mof2-1* strain [36], was used to probe interactions at the decoding center on the small subunit involving the aa-tRNA [37]. While the *scc2-4* mutant was hypersensitive to paromomycin (Fig 8D), as might be expected given the tendency for -1 frameshifting, inhibition of -1 frameshifting with anisomycin [35] did not rescue the growth defect observed in the mutant (S10 Fig). The failure of anisomycin to rescue the growth of the *scc2-4* mutant probably reflects the fact that Scc2 contributes to growth in multiple ways. Overall, our results are consistent with Scc2 normally promoting the production of rRNA and translational fidelity.

## Discussion

In this report, we characterized the biological processes controlled by Scc2, and show that Scc2 promotes the normal expression of hundreds of genes. With the notable exception of the centromeric region, Scc2 binding at the promoter regions of ribosomal protein genes, snoDNAs, and tDNAs was reduced in the *scc2-4* mutant. At the snoDNAs in particular, lower levels of Scc2 recruitment correlated with lower levels of RNA; and at these genes, Scc2 recruitment was partially dependent on the Paf1 transcription complex. We show that lower levels of H/ACA snoRNAs correlate with reduced pseudouridylation in the *scc2-4* mutant and decreased translational fidelity. Therefore, Scc2 promotes a gene expression program that supports translational fidelity.

### Scc2 coordinates events during RNA transcription and modification

Studies in human, fly, and yeast genomes show that Scc2 binds to the promoter regions of active genes [5, 11, 14, 38]. However, how Scc2 contributes to the expression of active genes is not well understood. From ChIP-seq analysis, we observed that Scc2 not only binds to the promoter regions of genes, but also in the gene body of snoRNA and tRNA genes (Fig 2). Binding of Scc2 to both promoter and genes bodies (in particular the snoDNAs) suggest that its role in regulating transcription might not be restricted to initiation. In support of this hypothesis, we observed that Paf1, a protein required for transcription elongation by RNA Pol II [29], is significantly reduced in the *scc2-4* mutant. Studies in budding yeast have shown that Paf1 is recruited to the promoter and coding regions of genes and aids in the processing of snoRNA transcripts [30]. The observation that Scc2 and Paf1 recruitment depend on each other at snoDNAs suggests the hypothesis that Paf1 and Scc2 together promote production of snoRNAs. This hypothesis is further supported by the observation that *paf1Δ* mutants have translational fidelity defects similar to the *scc2-4* mutant. Paf1 levels are reduced in the *scc2-4* mutant and there is a global reduction in ChIP signal, suggesting this could contribute to differential gene expression. The translation of at least one subunit of the Paf1 complex could be compromised in the *scc2-4* mutant, and this could then impact the stability of the other subunits [39]. Alternatively, the mutation in Scc2 could directly impact the stability of the Paf1 complex, for instance, at shared binding sites. The cooperation of Paf1 and Scc2 could have wider implications in humans where the Paf1 complex is overexpressed in a wide range of cancers including prostate, breast, renal, and gastric cancers [40, 41].

Different groups of Scc2 target genes show different responses to the loss of binding. This could be due in part to other factors that operate at those gene groups. For instance, Scc2 binding to snoDNAs appears to depend on Paf1, but this is not the case at other regions of the genome such rDNA and RP genes. Instead, RP genes are expressed at higher levels with reduced Scc2 binding. The elevation may be due to competition between cohesin binding and RNA Pol II binding at these promoters. As the *scc2-4* mutant decreases cohesin loading, it may allow RNA Pol II more access (Patrick Grant, in prep).

### Mutations that affect cohesin result in differential gene expression and translation in budding yeast

The *scc2-4* mutant has translation defects. Some phenotypes are similar to what we previously reported in the acetyltransferase mutant, *eco1-W216G* [22], such as defects in nucleolar morphology, ribosome distribution, and rRNA production. In both cases these phenotypes could result from reduced cohesin and condensin at the rDNA. Therefore cohesin may be important for nucleolar structure and function [23]. However, a closer look reveals significant differences in both gene expression and translational processes in the two mutants. For example, only the *scc2-4* mutation is associated with reduced levels of box H/ACA snoRNAs and poor translational fidelity as well as elevated levels of ribosomal protein gene messages. The acetyltransferase mutation has reduced levels of ribosomal protein gene messages, increased levels of the stress induced transcription factor Gcn4, and a pronounced effect on translational efficiency. Reduced translational efficiency is observed in cells derived from RBS patients and in zebrafish models for CdLS, and these cohesinopathies models are partially rescued by stimulating translation [16, 42]. We speculate that the differences in gene expression between *scc2* and *eco1* mutants might be based in the molecular mechanisms by which they influence gene expression. While Scc2 may maintain nucleosome free regions and promote cohesin and condensin loading, Eco1 helps to maintain a high level of stable acetylated cohesin. Loss of function mutations in these two genes may have different effects on gene expression. This is consistent with mutations in these two genes causing distinct human syndromes.

### Cohesinopathies and ribosomopathies have similar characteristics

Ribosomopathies are diseases caused by mutations that affect ribosome biogenesis. One such disease is dyskeratosis congenita (DKC). Reduced pseudouridylation of RNA is part of the etiology of DKC, a ribosomopathy characterized by bone marrow failure, skin abnormalities and increased susceptibility to cancer [27, 43]. Mutations in a pseudouridine synthase that modifies ribosomal RNAs and other RNAs (*DKC1*/*CBF5*) cause some cases of DKC. Studies from X-DC patient lymphoblasts and fibroblasts show downregulation of Box H/ACA snoRNAs [44], a characteristic feature in *scc2-4* mutant. Interestingly, the *scc2-4* mutant mimics *cbf5* mutants which have an impaired ability to translate IRES containing genes. Our results suggest that the *scc2-4* mutation reduces rRNA production as well as modification, possibly in part by affecting the production of H/ACA snoRNAs, which are important for pseudouridylation, resulting in reduced translational fidelity. Reduced levels of some box C/D RNAs could also affect translation due to impaired methylation of rRNA. We previously reported that human Roberts syndrome (RBS) cells and zebrafish models of RBS have defects in ribosome biogenesis and protein synthesis [22, 42], suggesting that RBS is at least in part a ribosomopathy. Zebrafish models for CdLS also show reduced translation [16]. Our findings suggest a shared feature of cohesinopathies and ribosomopathies is defective translation. Translation might therefore serve as a therapeutic target for CdLS.

## Materials and Methods

### Yeast strains

All strains were derived from BY4741 (*MATa his3*Δ*0 leu2*Δ*0 met15*Δ*0 ura3*Δ*0*) (S1 Table). The *scc2-4* mutations was originally isolated by the Nasmyth laboratory [1]. We constructed *scc2-4* mutant strains in the laboratory by PCR amplifying the mutation fused to a drug resistance marker from yeast genomic DNA, transforming the PCR product into the desired strain background, and isolating temperature sensitive colonies. The presence of the mutation was confirmed by sequencing.

### Measurement of RNA synthesis in vivo

Ribosomal RNA production was examined as previously described with some modifications [45]. Briefly, duplicate cultures of WT and *scc2-4* mutant strains were grown to mid-log phase (OD_600_ = 0.3) in SD-ura medium supplemented with 6.7 ng/μl uracil. ^3^H-uridine (5 μCi) was added to 500 μL of each culture and incubated at 30°C for 5 min with aeration. Samples were treated with 2.5 mL 10% trichloroacetic acid (TCA) and 2.5 mg/ml of uridine. After filtration through nitrocellulose, each membrane was washed with 5% TCA, dried, and counted in a Beckman LS 6500 multipurpose scintillation counter.

### Metabolic labeling-protein

WT and *scc2-4* strains were grown till mid-log phase YPD+CSM medium. Cells were pelleted, washed in PBS and resuspended in a similar volume of pre-warmed SD-met medium. Aliquot for zero time point was taken. Samples were then supplemented with 27.5 μCi ^35^S-methionine and 1mg/ml unlabeled-methionine. Samples were withdrawn at 15 min intervals for 2 hr. Amount of incorporated ^35^S-methionine in proteins was measured by adapting Kang and Hershey approach [46]. Briefly, cells were lysed in 1.8 N NaOH buffer containing 0.2 M β-mercaptoethanol. Proteins were precipitated with hot 10% trichloroacetic acid and precipitates washed twice in acetone. The precipitates were dissolved in 1% SDS and boiled for 10 min. Aliquots of samples were counted for ^35^S-methionine incorporation using a scintillation counter.

### rRNA processing

WT and *scc2-4* mutants strains were grown in SD-methionine medium to mid-log phase (OD_600_ = 0.3) Cells were pulse-labeled for 2 min with 250 μCi/ml ^3^H-methylmethionine and chased with 5 mM cold methionine. Samples were removed at 0, 2, 5 and 15 min intervals and flash-frozen in liquid nitrogen. RNA was extracted from cells, run on 1% formaldehyde agarose gel, transferred to a Hybond-N^+^ nylon membrane (GE Healthcare), and detected by autoradiography.

### RNA sequencing

WT and *scc2-4* mutant strains were grown in triplicate to mid-log phase (~OD_600_ = 0.8) in YPD+CSM medium. Cells were pelleted and RNA was extracted. RNA integrity was checked following isolation using an Agilent Technologies 2100 Bioanalyzer and a 1.2% formaldehyde agarose gel. Samples were depleted of ribosomal RNA using the Epicentre Ribo-zero Gold kit (human/mouse/rat, cat# RZG1224) according to manufacturer’s protocol. Libraries for sequencing were prepared using the Illumina Truseq RNA library preparation kit (cat# RS-930-2001) according to the manufacturer’s protocol. Libraries were pooled and run on an agarose gel, size selected from 200-400bp (with adapters), and run on the Hi-seq. Reads were mapped to saccCer2 using tophat to generate bam alignment files. Gene coordinates from ensembl biomart were then iterated over to count the number of reads mapping to each feature. These counts were then used to generate gene expression coefficients and statistics using the DESeq package in R. The GO term enrichment analysis was carried out using the hypergeometric test as implemented in the GeneAnswers package from Bioconductor. For the metagene analysis, reads were extended to 150 base pairs before calculating coverage. Coverage was adjusted to reads per million. For a group of genes, RPM coverage was extracted for 600 bp on either side of the TSS and the values were averaged per base pair for the group.

### Splicing analysis

The generated fastq files from RNA sequencing were aligned using tophat [47] against the *S*. *cerevisiae* genome assembly EF4 and gene annotation from the Ensembl release 69 (Oct. 2012) with the default options. Between 17.4M and 26.0M reads were aligned with 82.2% to 85.1% uniquely mapped reads per library. Splicing efficiency (expressed as Percent Spliced Out or PSO) was measured by computing the mean nucleotide coverage over every spliced junctions divided by the mean nucleotide coverage for reads falling within the exonic splicing unit (i.e. counting all the reads overlapping within the two exons flanking the splice junctions and dividing by the length of the two exons). Significant changes in PSO were computed by performing ANOVA for each splicing unit. The computed probabilities of the difference between the means were adjusted for multiple testing using the Benjamini and Hochberg method.

### ChIP

Genome-wide chromatin immunoprecipitation sequencing (ChIP-seq) was performed as previously described [48]. Briefly, yeast strains were cultured in duplicate in YPD to mid-log phase (OD_600_ = 0.8). Strains were crosslinked for either 15 min or 2 hrs (for Scc2 ChIP) with 1% formaldehyde at room temperature with occasional swirling. Crosslinnking was quenched with 2.5M Glycine. Cultures were pelleted, washed with cold PBS, and resuspended in PBS and placed at 4°C overnight. Cells were spheroplasted with 2.5 mg/ml zymolyase. Spheroplasts were washed and sonicated in SDS lysis buffer (1 mM PMSF, 0.μg/ml pepstatin A, 0.6 μg/ml leupeptin) to between 300–1000 bp in length. Chromatin extracts were diluted in immunoprecipitation (IP) buffer, debris was pelleted, and the supernatant was decanted into conical tubes. This chromatin solution was aliquoted for IPs. Chromatin extracts were immunoprecipitated overnight with antibody at 4°C followed by the addition of IgG beads. Beads were washed in several steps, and DNA was recovered in 1% SDS/0.1 M NaHCO_3_ elution buffer. Crosslinking was reversed by incubation at 65°C overnight, followed by protease treatment, phenol chloroform extraction, and ethanol precipitation of the recovered DNA. IPs were performed in duplicate for each sample. Sequence libraries were constructed and validated. Reads were mapped to saccCer2 using tophat to generate bam alignment files. Antibodies used for ChIP experiments are as follows; Myc antibody from Cell signaling (Cat #2276), HA antibody (12CA5) from Roche and RNA Pol II CTD4H8 antibody from Millipore (4 ul per 1mg protein IP).

### Microscopy and cytometry

Accumulation of ribosomal protein GFP fusion proteins in strains was measured with spinning disc confocal microscopy (Zeiss). The GFP intensity in WT and *scc2-4* mutant strains was quantified as previously described [22]. Briefly, peak GFP fluorescence intensity in each cell was calculated by measuring the pulse height in the cell using a B1 detector (525/50 emission). For each sample approximately 10,000 cells were measured. To quantify the distance the empirical distribution function of two samples, the Kolmogorov-Smirnov (KS) statistic was used. This statistic enables us to calculate the distance between two biological replicates (same genotype) and between samples with different genotypes since the distribution of fluorescence intensity among GFP positive cells is non-Gaussian. Using KS-distance enables us to determine whether the distance between WT and mutant samples is significantly greater than between replicates of the same genotype.

### Luciferase assays

The IRES and frameshifting assays were performed as previously described [33, 49]. Briefly, yeast strains were transformed with the indicated reporter plasmids. To examine IRES activity, overnight cultures of strains harboring CrPV IGR IRES and mutant cc-gg constructs were subcultured in SD-leu medium to mid-log phase (approx. OD_600_ = 0.5 to 1.0). Cells were lysed with 100 μl 1X passive lysis buffer (PLB) for 2 min. A dual luciferase assay kit (Promega) was used to measure luminescence according to the manufacturer’s protocol. IRES activity was measured using Firefly/Renilla ratio normalized to the Firefly/Renilla ratio of the wild type strain.

The frameshifting and readthrough assays were conducted with dual luciferase reporters [33]. Translational fidelity assays employed the dual-luciferase reporter system using pJD375 (the 0-frame control), pJD377 (containing the Ty1 +1 PRF signal), pJD376 (containing the yeast L-A virus -1 PRF signal). Luciferase activities were determined as previously described [50, 51]. To measure frameshifting, firefly/renilla luciferase ratio generated from the 0-frame control reporter (pJD375) was divided into that from the frameshifting signal-containing constructs (pJD377 and pJD376) and multiplied by 100% to obtain the frameshifting efficiencies for each construct. For the readthrough assay, overnight cultures of strains transformed with dual luciferase construct were subcultured and grown to mid-log phase, pelleted, lysed with 1X passive lysis buffer and dual luciferase assay performed in triplicates according to manufacturer’s protocol (Promega). Percentage readthrough was then calculated as previously described [33]. Readthrough occurred at a stop codon if firefly luciferase was translated following the Renilla luciferase ORF. Values obtained from firefly luciferase were normalized to Renilla luciferase activity as an internal control. The value obtained was then divided by the luciferase activity normalized to Renilla luciferase from a reporter with no stop codon and theoretically could be 100% readthrough for each reporter. Thus the percentage readthrough is expressed as Firefly/Renilla luciferase activity ratio divided by the firefly/Renilla luciferase activity ratio of the sense codon reporter and multiplied by 100.

### Antibiotic sensitivity

Growth and temperature sensitivity assays were performed using 10-fold serial dilutions of cells spotted onto the indicated medium and incubated at 30°C for 2–3 days. Maximal growth rates and sensitivity to translational inhibitors were determined using the TECAN machine in YPD medium containing 5 μg/mL anisomycin, or 800 μg/mL paromomycin.

### Western blotting

Western blot experiments were conducted using trichloroacetic acid (TCA) precipitation as previously described [52, 53]. Briefly overnight cultures were subcultured in YPD medium until mid-log phase (OD_600_ = 0.6). Cell were pelleted and washed 2 times in 20% TCA. Cells were resuspended in residual TCA and glass beads added. Cells were vortexed for 4 min at 3000 g and spun for 5 min at 14000 rpm. To each sample, 100 μl sample loading buffer was added and vortexed. Tris-HCl pH 8.8 or more was added and vortexed until the color changed to purple blue. Samples were boiled at 95°C for 5 min and loaded onto an SDS-PAGE gel.

### Pseudouridylation assay

Pseudouridylation assay was performed as previously described with a few modifications [54]. The experimental procedure is summarized below.

#### Reaction with CMC

Overnight cultures of yeast strains were subcultured and grown to mid-log phase (OD_600_ = 0.6). Cells were pelleted and RNA isolated from strains. RNA was dried and 8 μg RNA sample were treated with 30 μl of 0.17 M N-Cyclohexyl-N’-(2-morpholinoethyl)carbodiimide methyl-p-toluenesulfonate (CMC) in 50 mM Bicine, pH 8.3, 4 mM EDTA, and 7 M urea at 37°C for 20 min. The reaction was stopped with 100 μl of 0.3 M Sodium acetate (NaOAc) and 0.1 mM EDTA, pH 5.6 (buffer A) and 700ul 100% ethanol. Pellets were recovered, washed with 70% ethanol and dissolved in 100 μl buffer A, and reprecipitated with 300 μl 100% ethanol. Pellets were dried, and dissolved in 40 μl 50 mM Na_2_CO_3_, pH 10.4 and incubated at 37°C for 2 hrs. RNA was then precipitated with 100 μl buffer A and 700 μl of ethanol. Pellets were washed twice with 70% ethanol, dried and dissolved in 40 μl water.

#### Reverse transcription and gel analysis

RNA was reversed transcribed using 5’-end labeled primers. Hybridization was performed in a mixture containing 8 μg RNA, 4 * 10^5^ cpm/pmol 5’-labeled primer, and hybridization buffer (50 mM Tris-HCl, pH 8.5, 20 mM KCl) in a total volume of 5 μl. The mixture was incubated at 70°C for 3 min, transferred to a 37°C for 5 min, and then chilled on ice for 2 min or longer. The extension reaction was performed by adding 5 μl of a mixture containing 100 mM Tris-HCl, pH 8.5, 20 mM MgCl_2_, 20 mM DTT, 5 nM each of dATP, dGTP, dCTP, and dTTP, and 1.6 units of reverse transcriptase to the hybridization mixture and incubated at 37°C for 30 min. The extension reaction was stopped by adding 2 μl of a solution containing 0.5 mg/ml RNase A and 0.1 M EDTA, pH 6.5 and incubated at 37°C for 45 min. The sample mixture was then precipitated with 3 volumes of ethanol, dried and dissolved in 15 μl 75% Formamide, 1.4 mM Tris-HCl, pH 7.4, 1.4 mM EDTA, and 0.08% each of xylene cyanol FF and bromophenol blue. Samples were run on 8% polyacrylamide-7 M urea gel and detected with phosphoImager screens. Oligonucleotides for monitoring pseudouridylation were designed as previously described [55].

## Supporting Information

S1 Fig GO term analysis for the differentially expressed genes in the *scc2-4* mutant is shown by molecular function and cellular component. (A and B). GO term analysis of up-regulated genes in the *scc2-4* mutant showed enrichment for genes important for snoRNA binding, tRNA methyltransferase activity, nucleolar function and ribosome biogenesis (C and D). GO term analysis of down-regulated genes in the *scc2-4* mutant showed enrichment for genes important for oxidative reductase activity and respiratory chain.
(TIF)

S2 Fig The *scc2-4* mutant strain is sensitive to chloramphicol, an inhibitor of the mitochondrial ribosome. 10-fold serial dilutions of WT, *eco1-W216G* and *scc2-4* mutant strains from overnight cultures were grown at 30°C on YPD or YPD with 1μg/ml chloramphenicol. Plates were scanned after 2–3 days.
(TIF)

S3 Fig The *scc2-4* mutant strain shows reduced splicing. The percentage splicing index was calculated (described in Material and Methods) for WT, *scc2-4* mutant, and *eco1-W216G* mutant strains. Significant changes in splicing were computed by performing ANOVA for each splicing unit. Standard error is indicated for n = 3.
(TIF)

S4 Fig The rRNA processing rate is not affected in affected in the *scc2-4* mutant. rRNA processing was examined by growing strains to mid-log phase, labeling with ^3^H-methylmethionine for 2 min, chasing with 5 mM cold methionine and examining methylated rRNAs at 0, 2, 5, and 10 min time intervals. Equal amounts of radiolabelled RNA at each time point were compared by electrophoresis in a denaturing gel composed of 1% agarose and 16% formaldehyde. RNA was transferred to a HyBond-N^+^ nylon membrane, dried and visualized with autoradiography.
(TIF)

S5 Fig The *scc2-4* mutant strain shows defects in nucleolar morphology. The nucleolar morphology was examined by electron microscopy. Nucleoli were scored as aberrant if nucleoli did not have a compact crescent shape, were dispersed or undetectable. Scale bar represents 0.2 μm.
(TIF)

S6 Fig RNA Pol II and Tbf1 show normal or higher levels of recruitment, respectively, at snoDNAs in the *scc2-4* mutant strain. WT and *scc2-4* mutant strains were cultured as described in the method section and chromatin was extracted for ChIP. ChIP experiments were performed at least three times for each experiment. p-values were calculated using a student t-test. Standard errors is indicated for n = 3. (A) qPCR analysis shows enrichment of snoDNAs for RNA Pol II ChIP (CTD4H8, all forms, from Millipore) is similar in WT and mutant strains. ChIP performed without the addition of primary antibody serves as a negative control. (B) qPCR analysis shows enrichment of snoDNAs for Tbf1-Myc ChIP is similar or higher in the mutant strain relative to WT (α-Myc antibody, 9B11, Cell signaling). ChIP performed on an untagged strain serves as a negative control.
(TIF)

S7 Fig Recruitment of Scc2 at rDNA and RP genes not affected in the absence of Paf1. WT, *scc2-4*, and *paf1Δ* mutant strains were cultured in YPD medium to mid-log phase (~ OD_600_ = 0.5–0.8). Strains were crosslinked and chromatin extracted for ChIP. ChIP analysis was carried out for Scc2-Myc and Scc2^E534K^-Myc and Scc2-Myc *paf1Δ*. ChIP performed without the addition of primary antibody serves as a negative control. (A and B) qPCR analysis shows reduced enrichment for the indicated rDNA and RP genes relative to WT for Paf1 ChIP (α-HA antibody, 12CA5, Roche). (C and D) qPCR analysis shows normal enrichment for rDNA and RP genes for Scc2-Myc ChIP in the *paf1Δ* mutant strain relative to WT (α-Myc antibody, 9B11, Cell signaling). ChIP experiments were performed at least three times for each experiment. p-values were calculated using a student t-test. Standard error bars are indicated for n = 3. Significant values from the WT are indicated by an asterisk (p<0.05).
(TIF)

S8 Fig Protein synthesis is mildly affected in *scc2-4* mutant. (A) Polysome analysis of WT and *scc2-4* mutant strains. Polysome profiles from WT and *scc2-4* mutant strains were collected from cells grown to mid-log phase in YPD+CSM medium. Polysome profiling was done twice with similar results. No difference in polysome profiles between the WT and *scc2-4* mutant strains was observed. (B) ^35^S-methionine labeling was conducted to measure protein synthesis in WT and *scc2-4* mutant strains. Strains were grown to mid-log phase in SD-met supplemented with ^35^S-methionine. Cells were lysed, protein precipitated, and the amount of incorporated ^35^S-methionine measured with a scintillation counter. Standard error is indicated for n = 3. (C) The *scc2-4* mutant is sensitive to a sublethal concentration of cycloheximide. Overnight cultures of the WT and *scc2-4* mutant strains were serially diluted and spotted on YPD plates with or without cycloheximide and incubated at 30°C for 3 days.
(TIF)

S9 Fig Translational fidelity is reduced in the absence of *PAF1*. Dual luciferase reporters were used to measure translation. (A, top) Schematic diagram of CrPV IGR IRES containing reporter. Renilla luciferase is translated by a cap-dependent mechanism, and firefly luciferase synthesis requires cap-independent initiation mediated by the IRES. While cap-dependent translation is not affected, IRES-dependent translation is strongly inhibited in the *paf1Δ* mutant (p<0.05). (B, top) A dual luciferase reporter was used to monitor -1 frameshifting mediated by a sequence derived from the yeast L-A virus, and +1 frameshifting promoted by the yeast Ty1 sequence. In-frame renilla luciferase translation serves as a normalization control, and efficiencies were determined as previously described (Landry et al., 2009). p-values were calculated using student two-tailed t-test (asterisk indicates p<0.05). Standard error is indicated for at least three independent measurements.
(TIF)

S10 Fig Anisomycin does not rescue the growth defect in the *scc2-4* mutant. Serial dilutions of WT and *scc2-4* mutant strains were prepared in YPD medium with or without a low dose of Anisomycin (5 μg/ml). Cultures were aliquoted into 96 well plates and the growth rate was measured with a TECAN machine. The maximum growth rate was determined and the percentage growth rate in the presence or absence of anisomycin was calculated. p-values were calculated with student t-test. Standard error bars are indicated for n = 4.
(TIF)

S1 Table Strains used in this study. (DOCX)
