# Supplementary figures and images for: The SMC Loader Scc2 Promotes ncRNA Biogenesis and Translational Fidelity
Source: PLoS Genet. 2015 Jul 15;11(7):e1005308. doi: 10.1371/journal.pgen.1005308 (PMC4503661; doi:10.1371/journal.pgen.1005308)

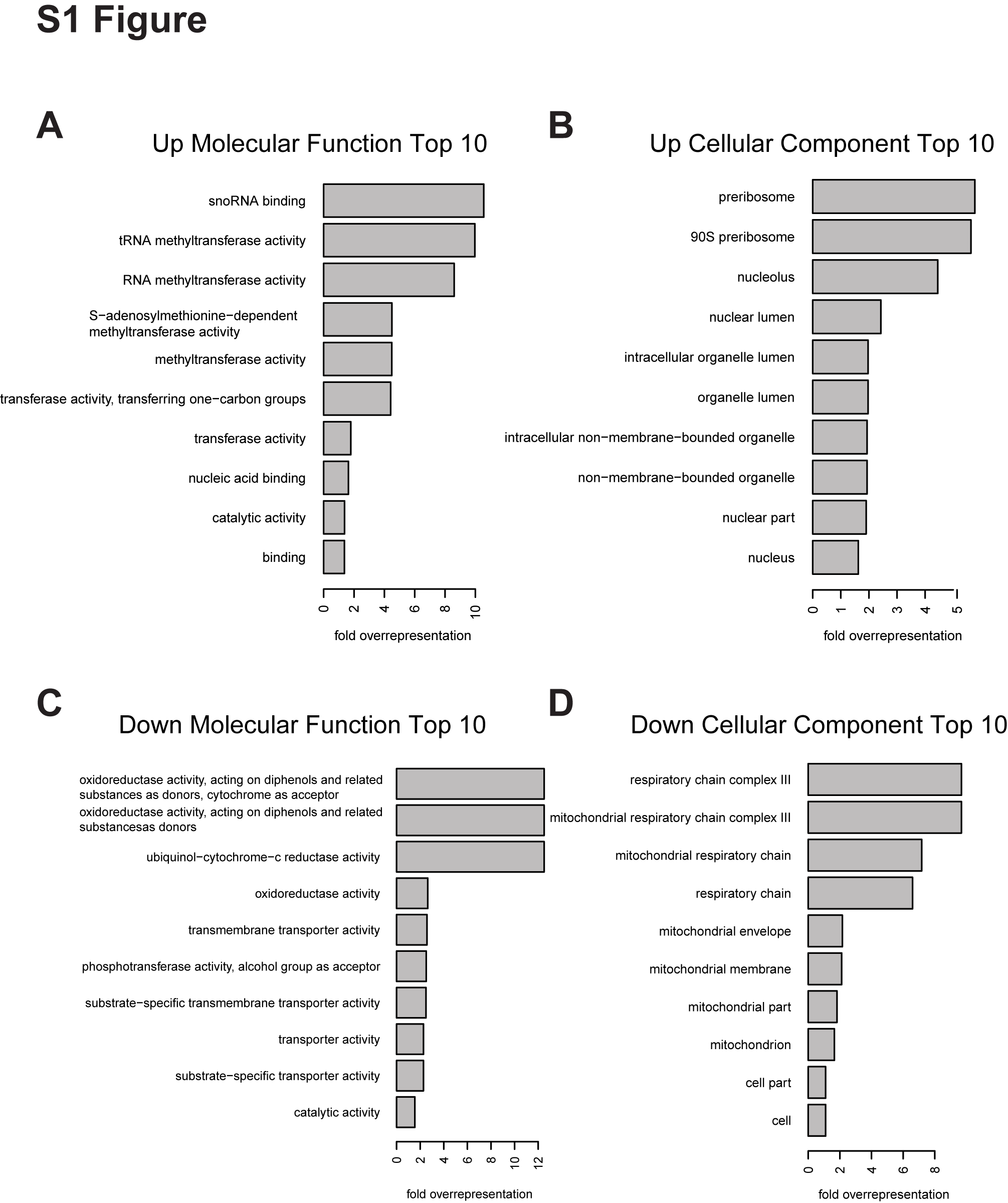

Supplement: S1 Fig — (A and B). GO term analysis of up-regulated genes in the scc2-4 mutant showed enrichment for genes important for snoRNA binding, tRNA methyltransferase activity, nucleolar function and ribosome biogenesis (C and D). GO term analysis of down-regulated genes in the scc2-4 mutant showed enrichment for genes important for oxidative reductase activity and respiratory chain. (TIF) [file pgen.1005308.s001.tif]

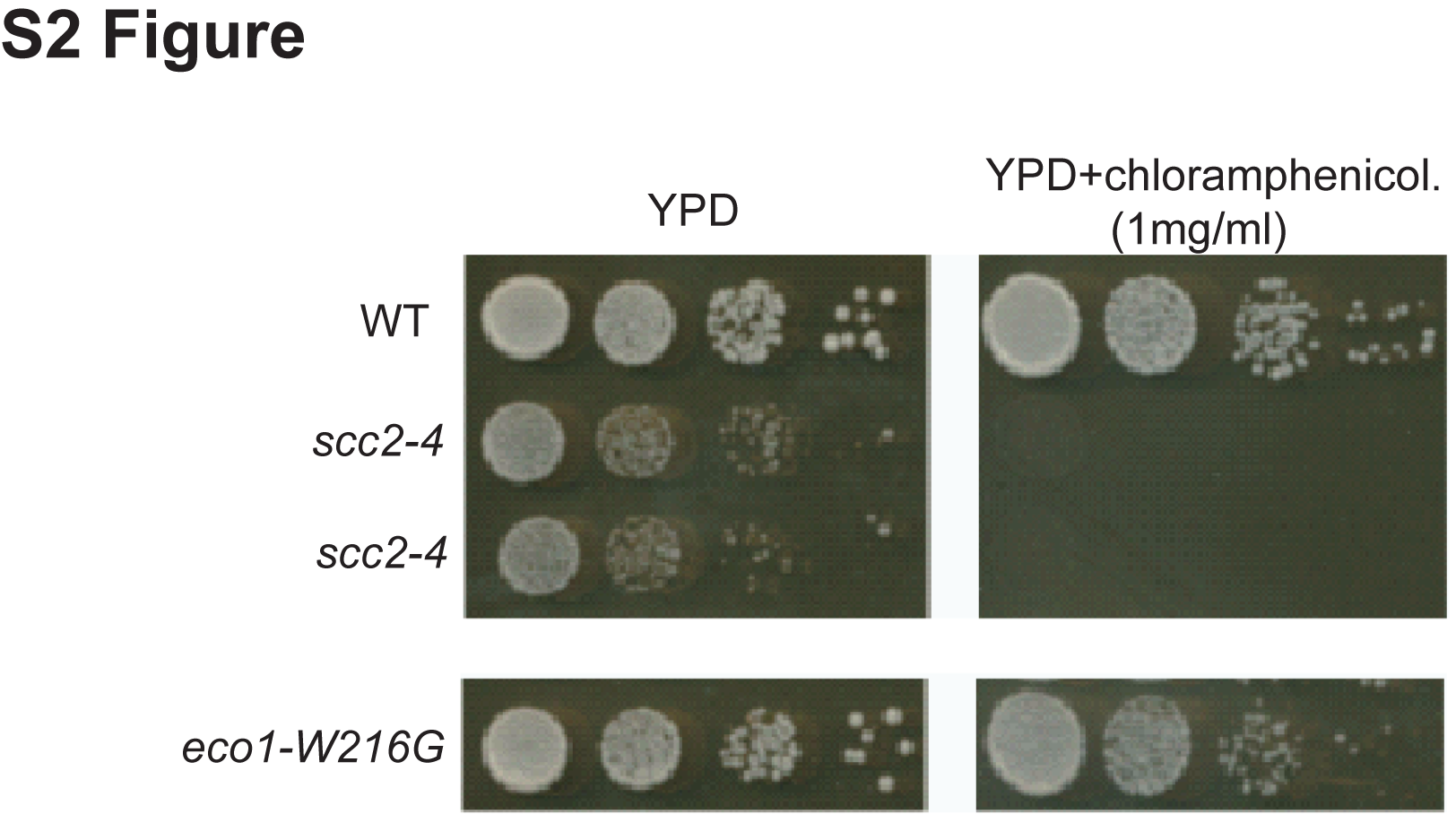

Supplement: S2 Fig — 10-fold serial dilutions of WT, eco1-W216G and scc2-4 mutant strains from overnight cultures were grown at 30°C on YPD or YPD with 1μg/ml chloramphenicol. Plates were scanned after 2–3 days. (TIF) [file pgen.1005308.s002.tif]

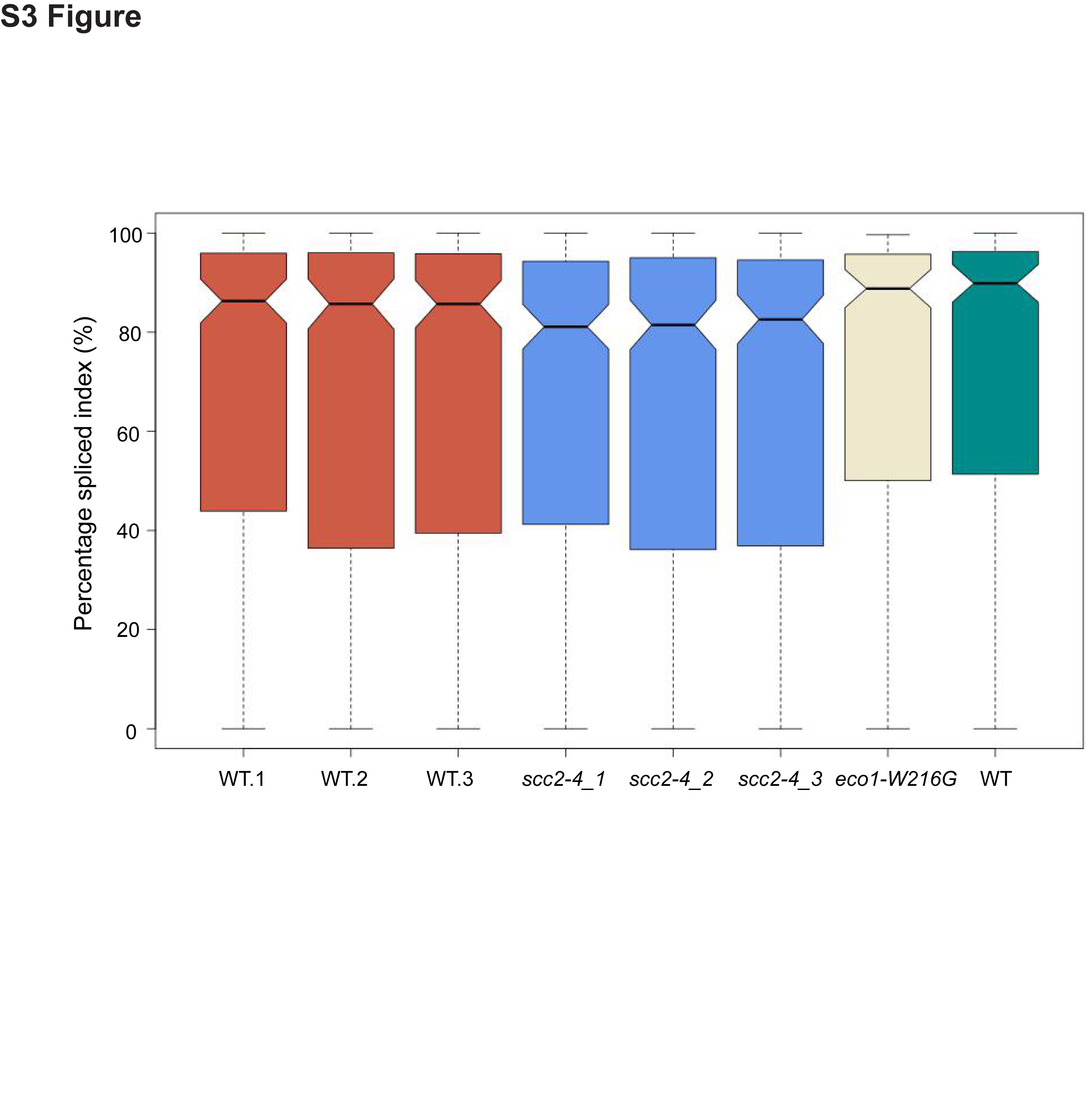

Supplement: S3 Fig — The percentage splicing index was calculated (described in Material and Methods) for WT, scc2-4 mutant, and eco1-W216G mutant strains. Significant changes in splicing were computed by performing ANOVA for each splicing unit. Standard error is indicated for n = 3. (TIF) [file pgen.1005308.s003.tif]

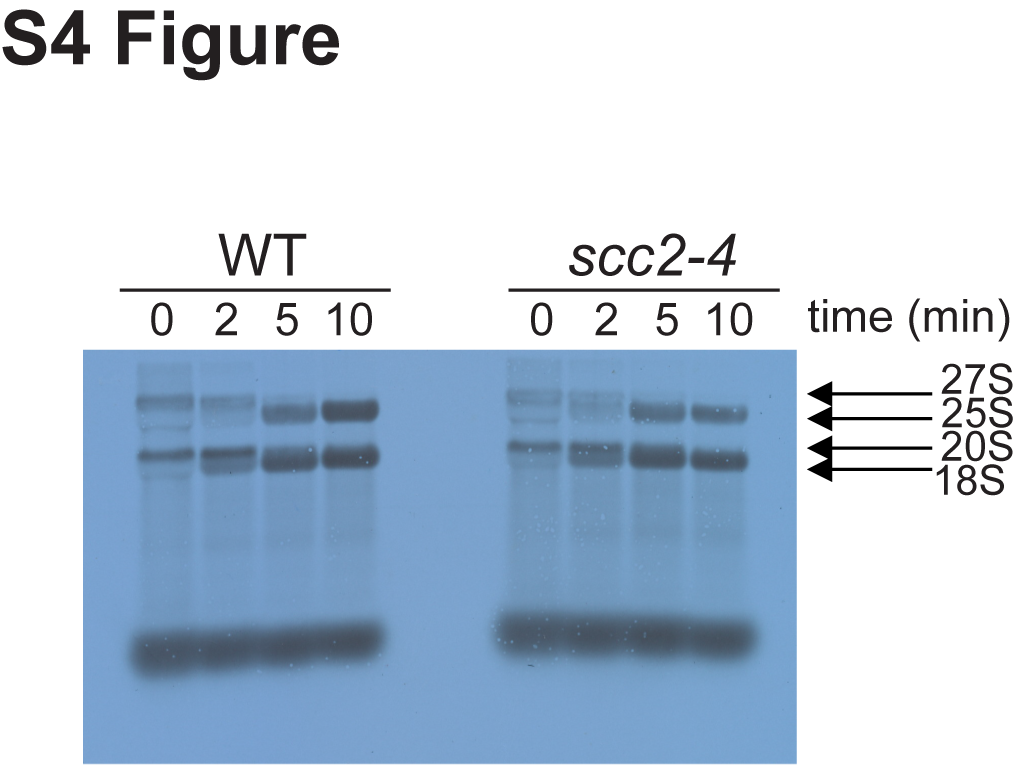

Supplement: S4 Fig — rRNA processing was examined by growing strains to mid-log phase, labeling with 3H-methylmethionine for 2 min, chasing with 5 mM cold methionine and examining methylated rRNAs at 0, 2, 5, and 10 min time intervals. Equal amounts of radiolabelled RNA at each time point were compared by electrophoresis in a denaturing gel composed of 1% agarose and 16% formaldehyde. RNA was transferred to a HyBond-N+ nylon membrane, dried and visualized with autoradiography. (TIF) [file pgen.1005308.s004.tif]

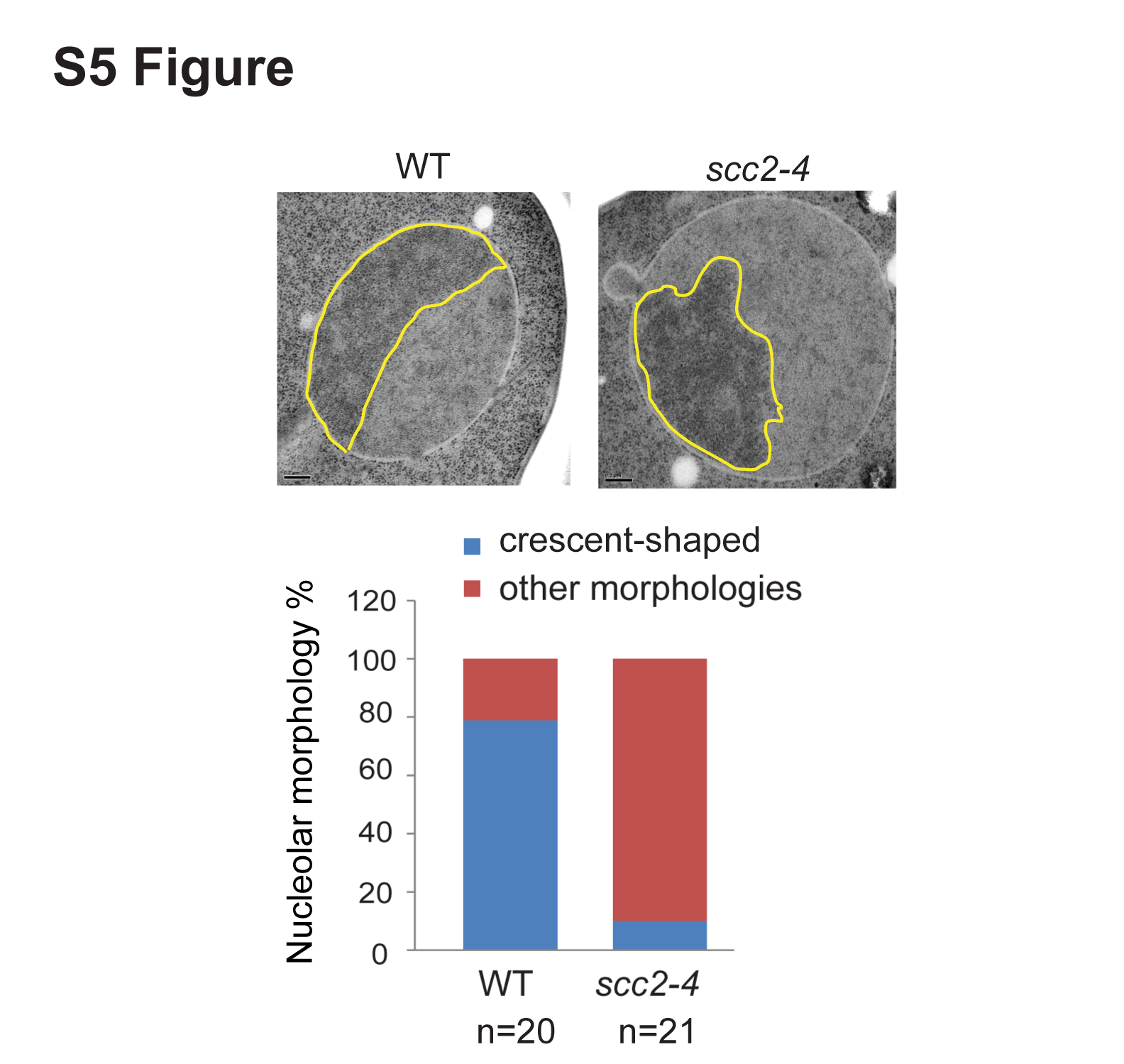

Supplement: S5 Fig — The nucleolar morphology was examined by electron microscopy. Nucleoli were scored as aberrant if nucleoli did not have a compact crescent shape, were dispersed or undetectable. Scale bar represents 0.2 μm. (TIF) [file pgen.1005308.s005.tif]

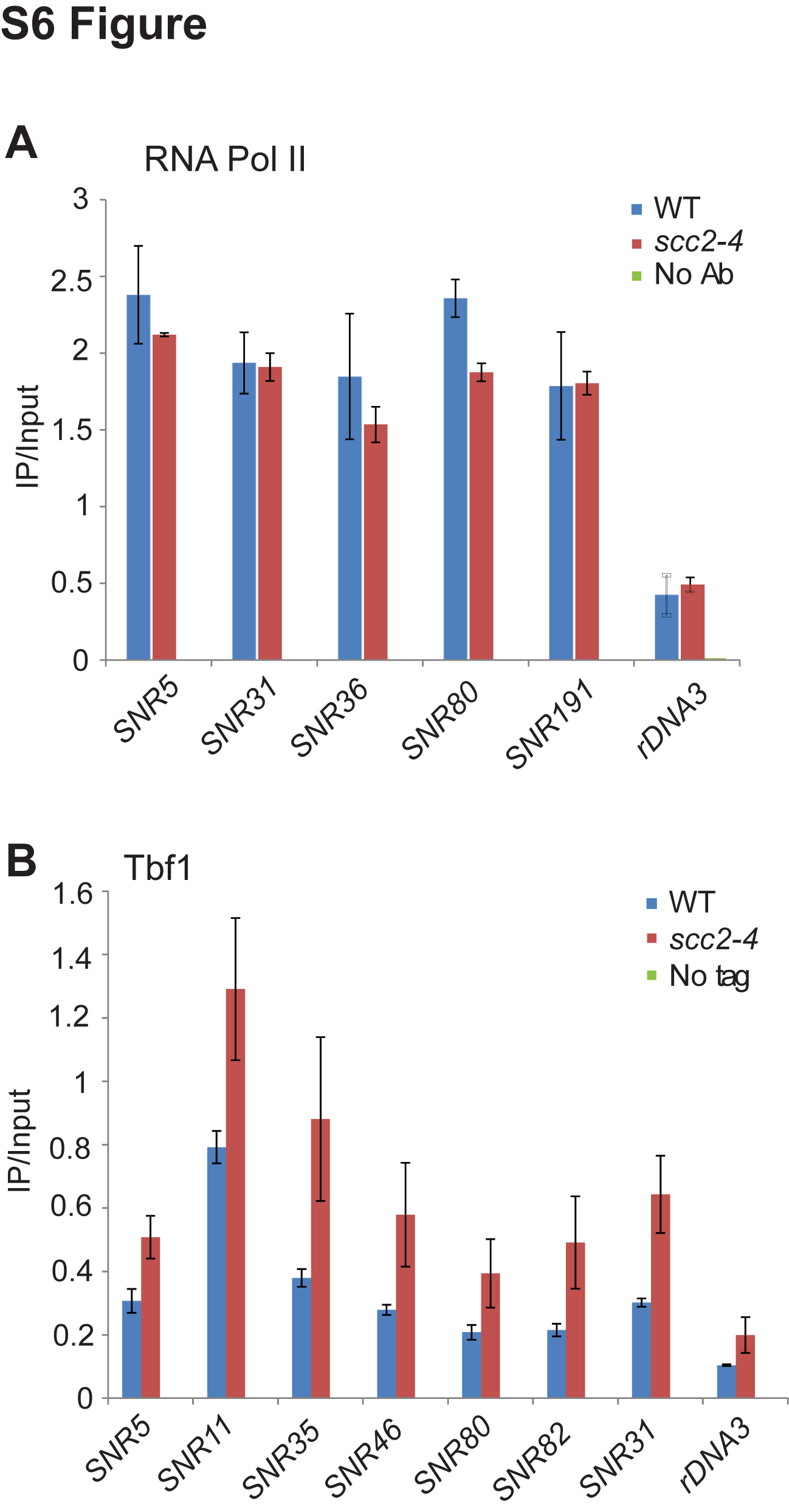

Supplement: S6 Fig — WT and scc2-4 mutant strains were cultured as described in the method section and chromatin was extracted for ChIP. ChIP experiments were performed at least three times for each experiment. p-values were calculated using a student t-test. Standard errors is indicated for n = 3. (A) qPCR analysis shows enrichment of snoDNAs for RNA Pol II ChIP (CTD4H8, all forms, from Millipore) is similar in WT and mutant strains. ChIP performed without the addition of primary antibody serves as a negative control. (B) qPCR analysis shows enrichment of snoDNAs for Tbf1-Myc ChIP is similar or higher in the mutant strain relative to WT (α-Myc antibody, 9B11, Cell signaling). ChIP performed on an untagged strain serves as a negative control. (TIF) [file pgen.1005308.s006.tif]

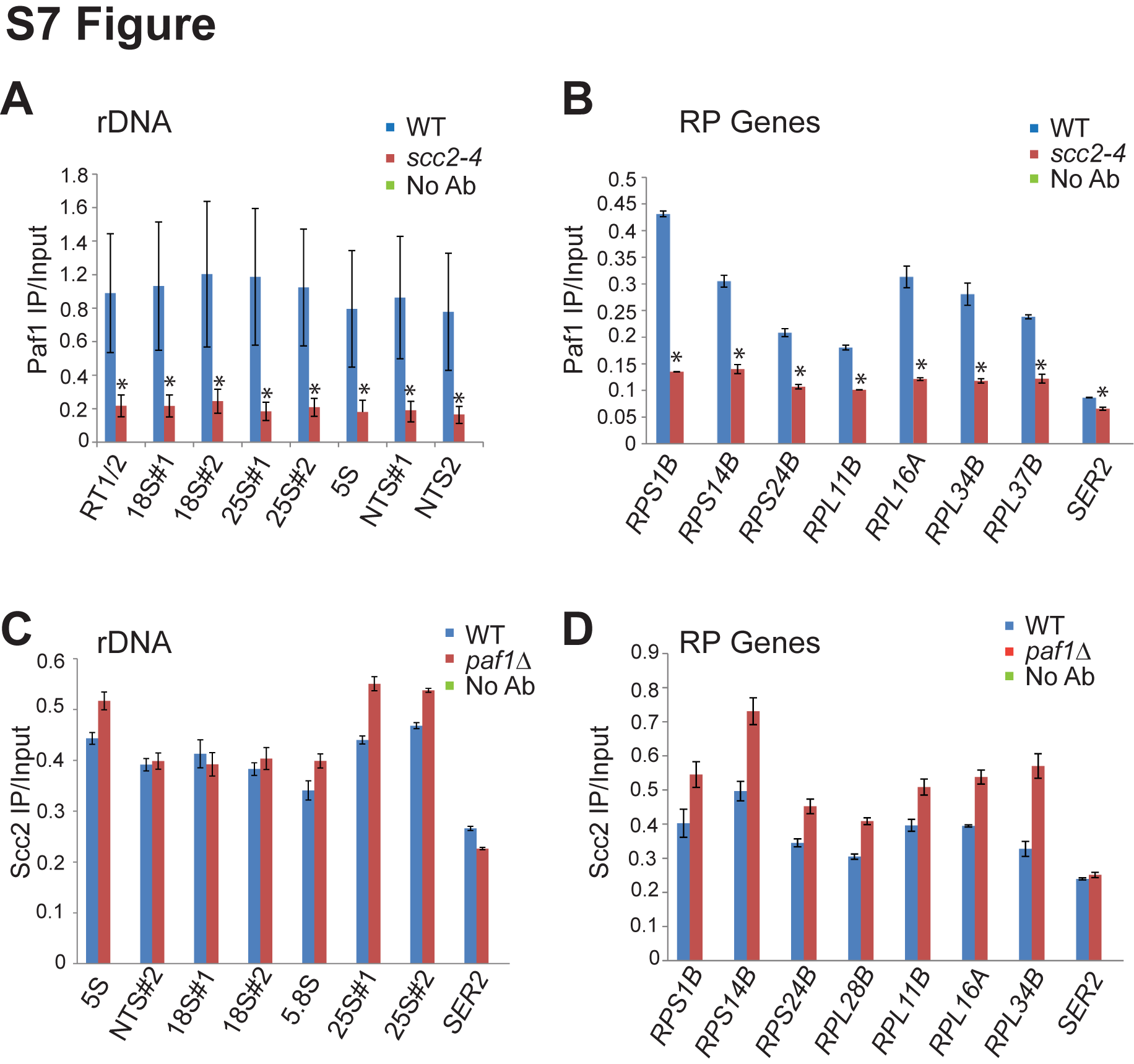

Supplement: S7 Fig — WT, scc2-4, and paf1Δ mutant strains were cultured in YPD medium to mid-log phase (~ OD600 = 0.5–0.8). Strains were crosslinked and chromatin extracted for ChIP. ChIP analysis was carried out for Scc2-Myc and Scc2E534K-Myc and Scc2-Myc paf1Δ. ChIP performed without the addition of primary antibody serves as a negative control. (A and B) qPCR analysis shows reduced enrichment for the indicated rDNA and RP genes relative to WT for Paf1 ChIP (α-HA antibody, 12CA5, Roche). (C and D) qPCR analysis shows normal enrichment for rDNA and RP genes for Scc2-Myc ChIP in the paf1Δ mutant strain relative to WT (α-Myc antibody, 9B11, Cell signaling). ChIP experiments were performed at least three times for each experiment. p-values were calculated using a student t-test. Standard error bars are indicated for n = 3. Significant values from the WT are indicated by an asterisk (p<0.05). (TIF) [file pgen.1005308.s007.tif]

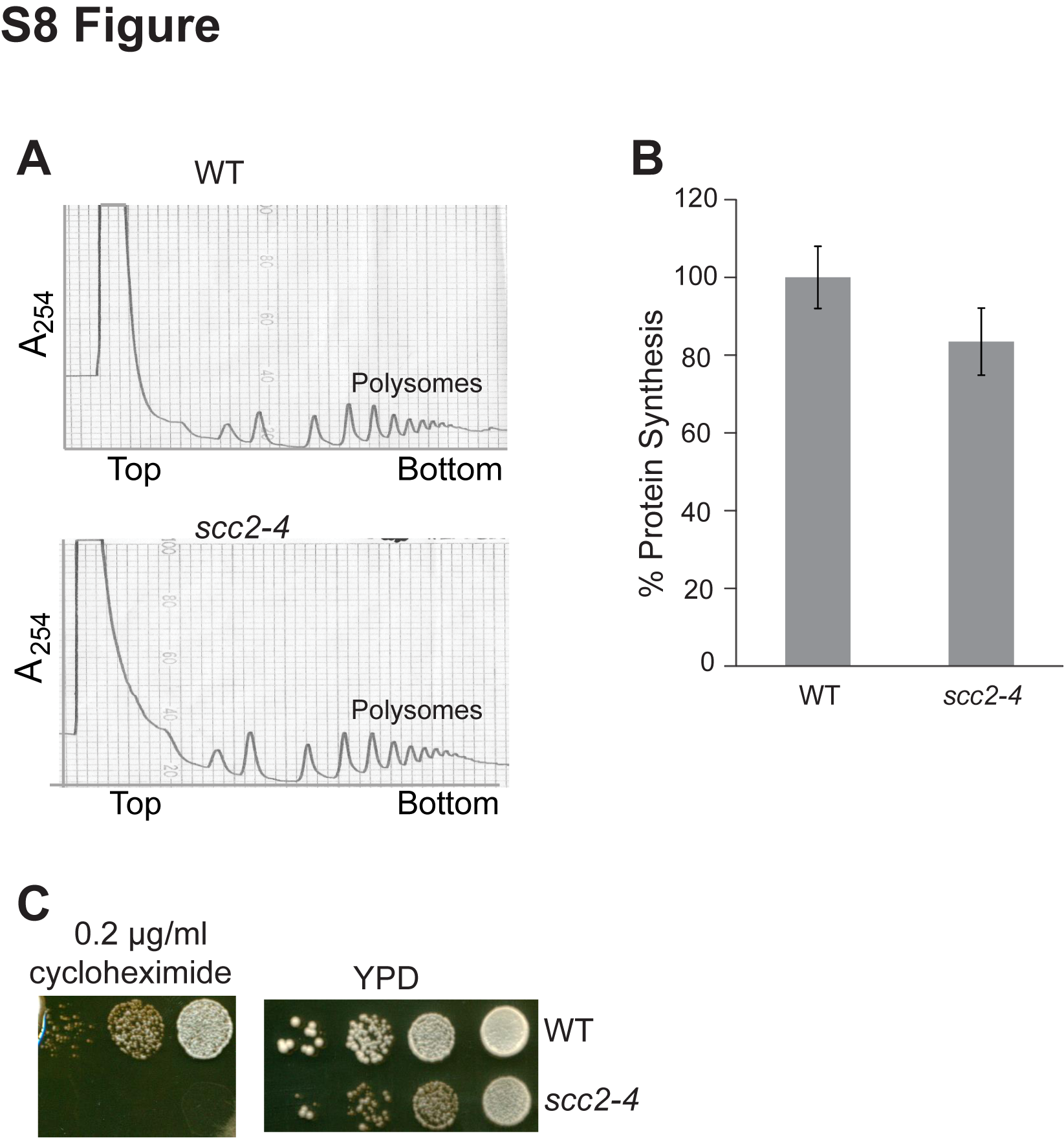

Supplement: S8 Fig — (A) Polysome analysis of WT and scc2-4 mutant strains. Polysome profiles from WT and scc2-4 mutant strains were collected from cells grown to mid-log phase in YPD+CSM medium. Polysome profiling was done twice with similar results. No difference in polysome profiles between the WT and scc2-4 mutant strains was observed. (B) 35S-methionine labeling was conducted to measure protein synthesis in WT and scc2-4 mutant strains. Strains were grown to mid-log phase in SD-met supplemented with 35S-methionine. Cells were lysed, protein precipitated, and the amount of incorporated 35S-methionine measured with a scintillation counter. Standard error is indicated for n = 3. (C) The scc2-4 mutant is sensitive to a sublethal concentration of cycloheximide. Overnight cultures of the WT and scc2-4 mutant strains were serially diluted and spotted on YPD plates with or without cycloheximide and incubated at 30°C for 3 days. (TIF) [file pgen.1005308.s008.tif]

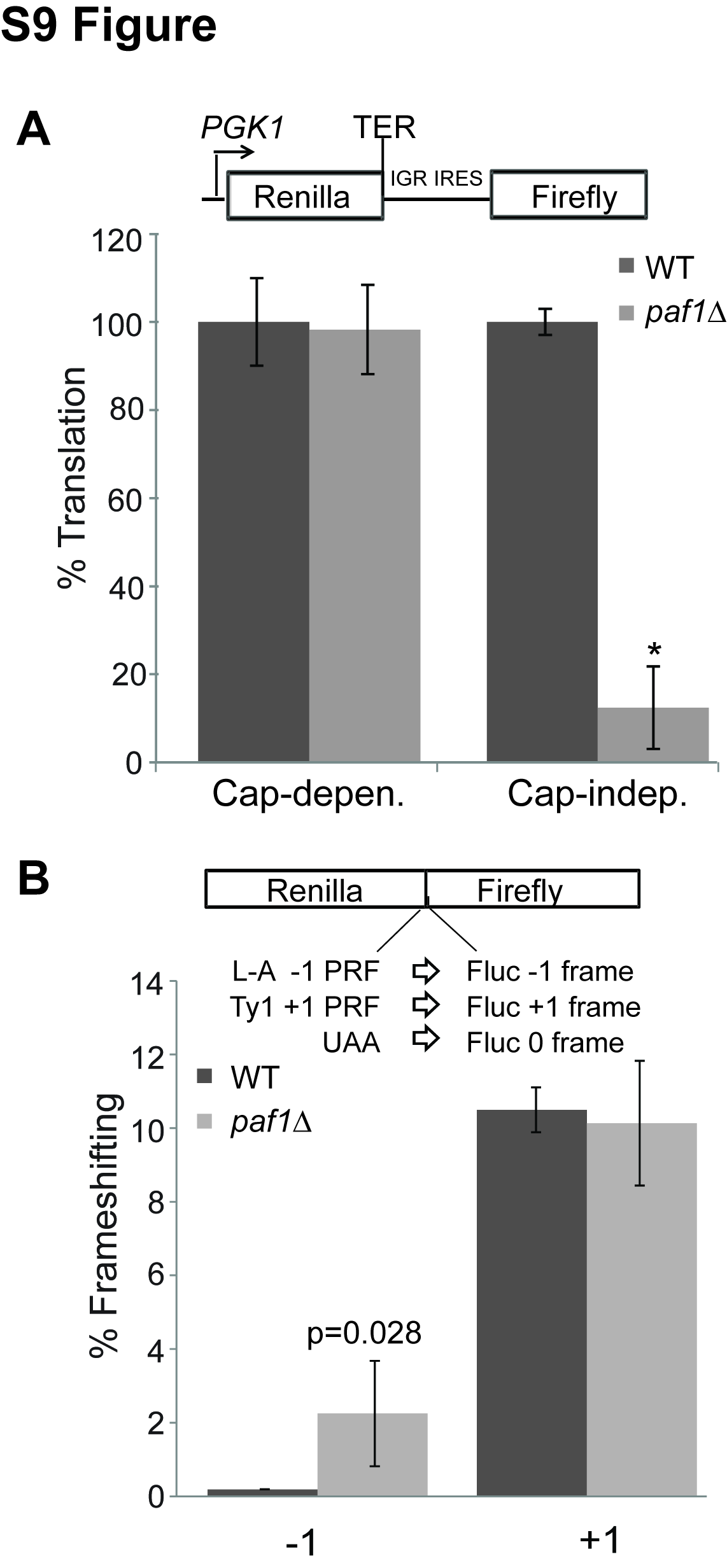

Supplement: S9 Fig — Dual luciferase reporters were used to measure translation. (A, top) Schematic diagram of CrPV IGR IRES containing reporter. Renilla luciferase is translated by a cap-dependent mechanism, and firefly luciferase synthesis requires cap-independent initiation mediated by the IRES. While cap-dependent translation is not affected, IRES-dependent translation is strongly inhibited in the paf1Δ mutant (p<0.05). (B, top) A dual luciferase reporter was used to monitor -1 frameshifting mediated by a sequence derived from the yeast L-A virus, and +1 frameshifting promoted by the yeast Ty1 sequence. In-frame renilla luciferase translation serves as a normalization control, and efficiencies were determined as previously described (Landry et al., 2009). p-values were calculated using student two-tailed t-test (asterisk indicates p<0.05). Standard error is indicated for at least three independent measurements. (TIF) [file pgen.1005308.s009.tif]

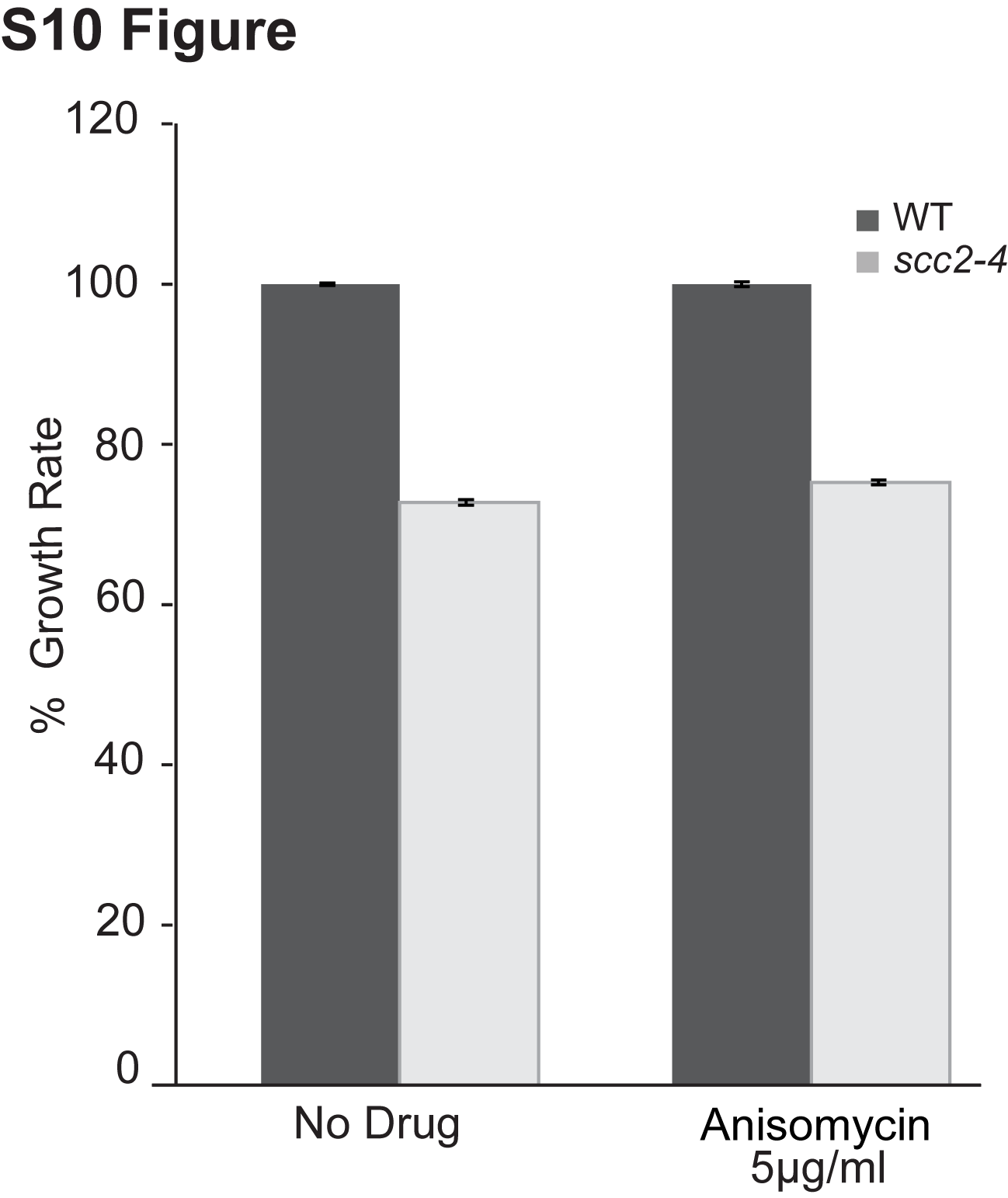

Supplement: S10 Fig — Serial dilutions of WT and scc2-4 mutant strains were prepared in YPD medium with or without a low dose of Anisomycin (5 μg/ml). Cultures were aliquoted into 96 well plates and the growth rate was measured with a TECAN machine. The maximum growth rate was determined and the percentage growth rate in the presence or absence of anisomycin was calculated. p-values were calculated with student t-test. Standard error bars are indicated for n = 4. (TIF) [file pgen.1005308.s010.tif]
