# Supplementary material for: The SMC Loader Scc2 Promotes ncRNA Biogenesis and Translational Fidelity
Source: PLoS Genet. 2015 Jul 15;11(7):e1005308. doi: 10.1371/journal.pgen.1005308 (PMC4503661; doi:10.1371/journal.pgen.1005308)
Supplement: S1 Table — (DOCX) [file pgen.1005308.s011.docx]

**S1 Table. Strains used in this study**

| Strain | Genotype | Reference |
| --- | --- | --- |
| BY4741 | MATa his3Δ leu2Δ0 met15Δ0 ura3Δ0 |  |
| MZ23 | MATa his3Δ leu2Δ0 met15Δ0 ura3Δ0 scc2-4::HYG | This study |
| MZ68 | MATa his3Δ leu2Δ0 met15Δ0 ura3Δ0 carrying pJD375 CEN URA plasmid | This study |
| MZ69 | MATa his3Δ leu2Δ0 met15Δ0 ura3Δ0 carrying pJD376 CEN URA plasmid | This study |
| MZ70 | MATa his3Δ leu2Δ0 met15Δ0 ura3Δ0 carrying pJD377 CEN URA plasmid | This study |
| MZ71 | MATa his3Δ leu2Δ0 met15Δ0 ura3Δ0 carrying pJD431 CEN URA plasmid | This study |
| MZ72 | MATa his3Δ leu2Δ0 met15Δ0 ura3Δ0 carrying pJD432 CEN URA plasmid | This study |
| MZ73 | MATa his3Δ leu2Δ0 met15Δ0 ura3Δ0 carrying pJD433 CEN URA plasmid | This study |
| MZ30 | MATa his3Δ leu2Δ0 met15Δ0 ura3Δ0 paf1Δ::NATMx6 SCC2-13MYC::KanMx6 | This study |
| MZ74 | MATa his3Δ leu2Δ0 met15Δ0 ura3Δ0 scc2-4::HYGMx6 carrying pJD433 CEN URA plasmid | This study |
| MZ75 | MATa his3Δ leu2Δ0 met15Δ0 ura3Δ0 scc2-4::HYGMx6 carrying pJD433 CEN URA plasmid | This study |
| MZ76 | MATa his3Δ leu2Δ0 met15Δ0 ura3Δ0 scc2-4::HYGMx6 carrying pJD433 CEN URA plasmid | This study |
| MZ77 | MATa his3Δ leu2Δ0 met15Δ0 ura3Δ0 scc2-4::HYGMx6 carrying pJD433 CEN URA plasmid | This study |
| MZ78 | MATa his3Δ leu2Δ0 met15Δ0 ura3Δ0 scc2-4::HYGMx6 carrying pJD433 CEN URA plasmid | This study |
| MZ79 | MATa his3Δ leu2Δ0 met15Δ0 ura3Δ0 scc2-4::HYGMx6 carrying pJD433 CEN URA plasmid | This study |
| MZ11 | MATa his3Δ leu2Δ0 met15Δ0 ura3Δ0 scc2-4::HYGMx6 carrying Rps2-GFP CEN LEU plasmid | This study |
| MZ13 | MATa his3Δ leu2Δ0 met15Δ0 ura3Δ0 scc2-4::HYGMx6 carrying Rpl25-GFP CEN LEU plasmid | This study |
| MZ12 | MATa his3Δ leu2Δ0 met15Δ0 ura3Δ0 scc2-4::HYGMx6 carrying Rps2-GFP CEN LEU plasmid | This study |
| MZ14 | MATa his3Δ leu2Δ0 met15Δ0 ura3Δ0 scc2-4::HYGMx6 carrying Rpl25-GFP CEN LEU plasmid | This study |
| MZ216 | MATa his3Δ leu2Δ0 met15Δ0 ura3Δ0 SCC2-13MYC::KanMx6 PAF1-3HA::HISMx6 | This study |
| MZ217 | MATa his3Δ leu2Δ0 met15Δ0 ura3Δ0 scc2-4-13MYC::KanMx6 PAF1-3HA::HisMx6 | This study |
| MZ219 | MATa his3Δ leu2Δ0 met15Δ0 ura3Δ0 SCC2-13MYC::KanMx6 CTR9-3HA::HisMx6 | This study |
| MZ220 | MATa his3Δ leu2Δ0 met15Δ0 ura3Δ0 scc2-4-13MYC::KanMx6 CTR9-3HA::HisMx6 | This study |
